# Functional Diversity of Riparian Woody Vegetation Is Less Affected by River Regulation in the Mediterranean Than Boreal Region

**DOI:** 10.3389/fpls.2020.00857

**Published:** 2020-06-24

**Authors:** Ivana Lozanovska, María Dolores Bejarano, Maria João Martins, Christer Nilsson, Maria Teresa Ferreira, Francisca C. Aguiar

**Affiliations:** ^1^Centro de Estudos Florestais, Instituto Superior de Agronomia, Universidade de Lisboa, Lisbon, Portugal; ^2^Landscape Ecology Group, Department of Ecology and Environmental Science, Umeå University, Umeå, Sweden; ^3^Department of Wildlife, Fish and Environmental Studies, Swedish University of Agricultural Sciences, Umeå, Sweden

**Keywords:** functional diversity, functional traits, functional richness, functional redundancy, riparian woody vegetation, streamflow regulation, boreal biome, mediterranean biome

## Abstract

River regulation may filter out riparian plants often resulting in reduced functional diversity, i.e., in the range of functions that organisms have in communities and ecosystems. There is, however, little empirical evidence about the magnitude of such reductions in different regions. We investigated the functional diversity patterns of riparian woody vegetation to streamflow regulation in boreal Sweden and Mediterranean Portugal using nine plant functional traits and field data from 109 sampling sites. We evaluated changes in mean plant functional traits as well as in indices of multidimensional functional traits, i.e., functional richness (FRic) and functional redundancy (FRed) within regions and between free-flowing and regulated river reaches. We found that regulation significantly reduced functional diversity in Sweden but not in Portugal. In Sweden, the increased magnitude of variations in water flow and water level in summer, the prolonged duration of extreme hydrological events, the increased frequency of high-water pulses, and the rate of change in water conditions were the likely main drivers of functional diversity change. Small riparian plant species with tiny leaves, poorly lignified stems, and shallow root systems were consistently associated with regulated sites in the boreal region. In Portugal, the similar functional diversity values for free-flowing and regulated rivers likely stem from the smaller streamflow alterations by regulation combined with the species legacy adaptations to the Mediterranean natural hydrological regimes. We conclude that streamflow regulation may reduce the functional diversity of riparian woody vegetation, but the magnitude of these effects will vary depending on the adaptations of the local flora and the patterns of streamflow disturbances. Our study provides insights into functional diversity patterns of riparian woody vegetation affected by regulation in contrasting biomes and encourages further studies of the functional diversity thresholds for maintaining ecosystems.

## Introduction

The alteration of streamflow regime is recognized as a key threat to many riverine plant species and compromises many functions and ecosystem services of rivers (Arthington et al., 2010; Tonkin et al., 2018). Dams, reservoirs and other infrastructures for river regulation are changing the natural streamflow regimes resulting in homogenization of river dynamics, reconstruction of riparian habitats and ultimately a reshaping of riparian vegetation (Lytle et al., 2017).

Riparian vegetation is particularly sensitive to fluctuations in flow and water-level and must cope with variations in inundation, water-stress and water currents (Bornette et al., 2008; Merritt et al., 2010). The adaptations of riparian species resulting from specific combinations of functional traits determine the fate of species under different flow conditions (Stromberg and Merritt, 2016). Since combinations of functional traits vary with environmental conditions, streamflow variations, among others, can filter out sensitive riparian plant species, leading to a reduction in the range of trait values and, ultimately, changes in functional diversity (Keddy, 1992; Hooper et al., 2005).

Functional diversity reflects the range of functions that organisms have in communities and ecosystems (Mouchet et al., 2010) and has been shown to respond to environmental filters (Bruno et al., 2016; Lozanovska et al., 2018b). Functional richness (FRic) and functional redundancy (Fred) represent two components of functional diversity that may be important for maintaining ecosystem functioning in response to stressors (Mori et al., 2013; Angeler and Allen, 2016). A combination of differences in the range of functional traits enables an ecosystem community to cope better with various environmental and/or anthropogenic disturbances by having at least one trait out of multiple which can mitigate the disturbance, thus maintaining ecosystem functioning (Tilman et al., 1997; Mouillot et al., 2013). Functional redundancy describes the situation when more than one species present similar species traits, and thus can compensate for species loss following stress (Walker, 1992). On the contrary, species loss in non-redundant communities leads to loss of traits or functions, further increasing ecosystem vulnerability to disturbances (Rosenfeld, 2002).

The critical attributes of the streamflow regime – magnitude, frequency, duration, timing and rate of change (Poff et al., 2007) – which govern riparian vegetation dynamics, vary with biogeographic, geomorphic, and climatic settings (Nilsson and Svedmark, 2002). In Mediterranean regions, rivers are influenced by the seasonality and variability of precipitation with dry summers and mild winters, and large interannual variability. Such rivers are naturally subjected to extremes, ranging between no or low flows to flash floods (Gasith and Resh, 1999). Temporary reductions in water availability play an important evolutionary role in adapting riparian vegetation to such conditions (Stromberg and Boudell, 2013). In the boreal region, however, most rivers and streams have permanent streamflow. The flow regime is driven by snow accumulation during winter when flows and water levels are at their lowest, and by melting of snow and ice during spring and early summer when floods reach their annual maximum levels (Woo et al., 2008). In the far northern latitudes, recurrent ice formation and ice jams can cause physical damage and physiological alterations in riparian plants (Nilsson et al., 2015).

Despite the diverse constraints of Mediterranean and boreal biomes in the physiology and physiognomy of riparian vegetation, altered seasonal and daily streamflow variations by dams are known to impair riparian ecosystems in general (Webb et al., 2013). In Portugal, damming has long been used to cope with the natural seasonality of precipitation and since the middle of the 20th century also to favor hydropower generation. In Sweden, due to the rapidly changing energy markets, rivers have been exploited for large-scale hydropower production since the early 20th century, with increased hydropeaking over recent decades (Ashraf et al., 2018).

Our main goal was to investigate if streamflow regulation in two biomes with a diverse legacy of plant adaptations and environmental constraints would lead to similar ecological patterns for functional diversity in riparian woody vegetation. We did this through an analysis of nine flow-related functional traits and two indices, i.e., FRic and Fred, in free-flowing and regulated rivers from both regions. We hypothesized that streamflow regulation would be reflected in the functional diversity patterns of riparian woody vegetation, affecting functional traits that are intolerant to streamflow regulation and reducing functional diversity in both regions. However, we expected that changes in functional diversity patterns would differ based on the streamflow regime and species’ natural adaptations to flow-related disturbances i.e., that biome can mediate the effect of streamflow regulation on functional diversity. Specifically, we asked the following questions: (i) how do functional diversity in the Mediterranean and boreal rivers change with regulation? (ii) which hydrological attributes affect functional diversity in Mediterranean and boreal ecosystems?

## Materials and Methods

### Study Area and Sampling Design

**FIGURE 1:**
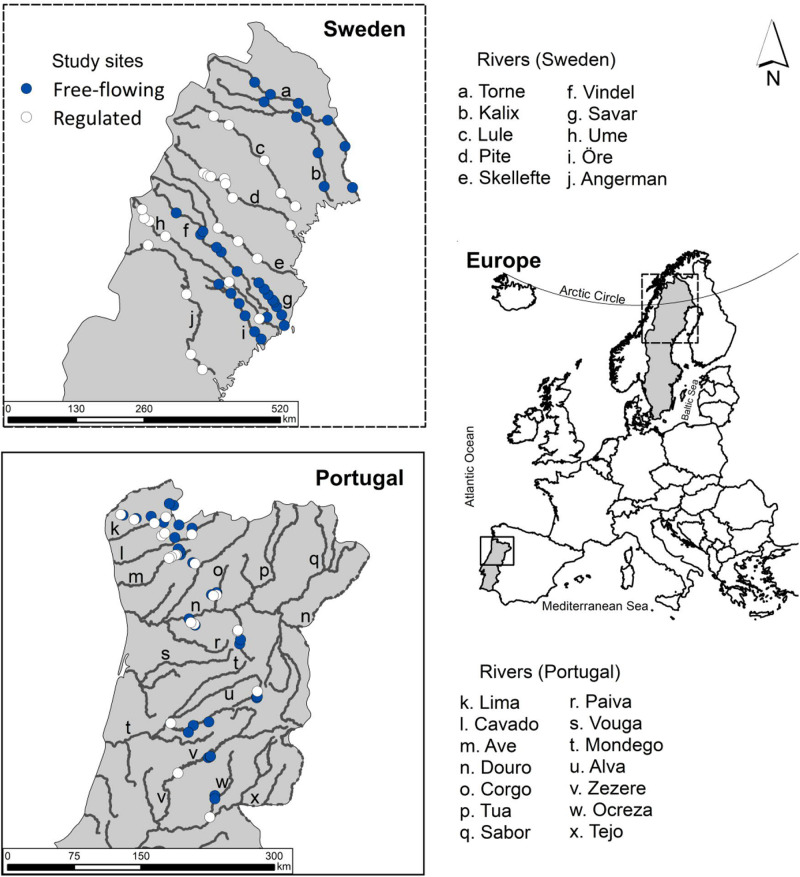
Location of the study sites in Sweden and Portugal. Filled circles represent sites at free-flowing rivers and unfilled circles represent sites at regulated rivers. Tributaries in Sweden are not shown.

Our study was undertaken in the north and central mainland of Portugal and in the boreal coniferous zone in northern Sweden (Figure 1). European Mediterranean and boreal biomes differ in climate, vegetation and fluvial dynamics (Table 1). Seasonality is the main factor controlling streamflow regimes in Mediterranean rivers, whereas rivers in the boreal biome are strongly influenced by frost formation, ice regimes and snowmelt. Hydromorphic disturbances are common in both biomes.

**TABLE 1:** Legacy effects that influence riparian woody vegetation in mediterranean and boreal biomes.

EU Mediterranean biome	EU Boreal Biome
***Location***	
Between the latitudes of 30° and 45°N	The arctic and subarctic (or boreal) latitudes between the North Pole to about 55°N
Surrounding the Mediterranean Sea, extends across 4,300,000 km^2^	Extends across 10 million km^2^ of the northern circumpolar region including Fennoscandia and large parts of North America and Russia
***Climate***	
Mediterranean climate (rainy winters; hot and dry summers)	Subarctic climate and humid continental climate
Average annual temperature 7.5–16.5°C	Average annual temperature 14–17°C in July, +1 to −14°C in January, February
Precipitation below 500 to 2800 mm	Precipitation average 300 to 1500 mm
	Snow as half of the annual precipitation
	Snowpack formations persistent for several months
***Geology and geomorphology***	
Composed by a pre-Mesozoic complex geologic unit – the Hesperic Massif (granite, schist and quartzite) in the inland area. Tertiary layers under Quaternary deposits at the western coastal fringe	Erosional and depositional landforms heavily formed by past periods of glaciation and present glacierisation Depositional glaciated and glacierised landscapes-moraines, eskers and drumlins
Low relief and extensive tributary networks	High relief cirques and U-shaped valley
***Soil formation and characteristics***	
Dissolution and leaching of calcium carbonate during winter and development of red dehydrated oxidized iron compounds-hematite, magnetite during summer	Soil transition from mineral soils (generally podzols) in upslope areas to organic soils (generally histosols) in the near-stream zone
***Streamflow patterns***	
Rivers are characterized by sequences of floods in autumn–winter and droughts in summer	Extensive permafrost thaw, ice regimes and snowmelt events determine the hydrological regimes
***Vegetation***	
Sclerophyllous and evergreens due to soil infertility and as an additional defense against herbivory; wetter range with deciduous species highly responsive to flooding and hydrological dynamics	Evergreens due to a longer photosynthetic season and nutrient poor substrata
***Biomass production and decomposition***	
Leaf litter decomposition slower than in temperate areas Prolonged riparian inputs to the streams (rather than concentrated in autumn)	Biomass production variable with flood frequency and duration (increasing towards lower riparian elevations)
Low accumulation of organic matter	Accumulation of high levels of organic matter
***Riparian species adaptations***	
Adapted to natural water variability (shorter canopy height, higher colonization rates)	Elevated biological activities during growing season and depressed/dormant during the frozen period
***Ecosystem invasibility***	
High susceptibility to invasion	Few occurrences of exotic species; absence of invasive species
***Main threats in fluvial systems***	
Damming	Damming
Water abstraction for irrigation	Ditching
Land-use and land-cover change	
Plant invasions	
Fire	

*References are given in Supplementary Table S9.*

The Portuguese study sites represent a Mediterranean climate with hot, dry summers and mild, wet winters. Riparian woodlands comprise heterogeneous assemblages dominated by winter deciduous species. Alder woodlands composed of the black alder (*Alnus glutinosa*) and gray willow (*Salix atrocinerea*), with *Rubus* spp. on the edge of the riparian zone and the Portuguese tussock sedge (*Carex paniculata* subsp. *lusitanica*) on and along the river channel. In perennial rivers with seasonally irregular flows, Ash woodlands occur and are dominated by narrow-leaved ash (*Fraxinus angustifolia*) and an Iberian willow (*Salix salviifolia*). Along the perennial rivers, the riparian shrub strata frequently include *Crataegus monogyna* and *Rubus* spp. In torrential rivers, *Salix salviifolia* commonly borders rivers and streams. Riparian forests in Portugal are usually constrained by the agricultural and forestry land-uses adjacent to rivers. In regulated reaches, occurrence of alien invasive species, such as *Acacia* spp. and *Arundo donax* is common. While species richness is similar between the free-flowing and regulated river reaches, the regulated river reaches are more fragmented and narrower, and are lacking the natural spatial zonation of the riparian communities (Aguiar et al., 2016). For instance, willow species can invade rivers downstream of dams in almost monospecific stands.

The Swedish study sites have a cold-temperate climate. The riparian vegetation along the free-flowing rivers is distinctly vertically zoned, from forest communities at the top with *Pinus sylvestris* and *Alnus incana* among the dominant tree species, to shrub vegetation of predominantly *Salix* spp., to herbaceous communities with *Carex* spp. and amphibious species such as *Ranunculus reptans* at the bottom. The riparian vegetation along the regulated rivers generally lacks the distinctive zonation. Instead, it can be separated into a narrow strip without clear riparian plant dominants close to the high-water level, and below this is a sparse occurrence of amphibious species such as *Ranunculus reptans* and *Subularia aquatica*.

Portuguese sampling sites include small and medium-sized rivers with an average mean monthly flow of 7 m^3^/s. The dataset included 30 slightly impaired river reaches (hereafter “free-flowing”), from the national reference database of the Portuguese Environment Agency (Agência Portuguesa do Ambiente, APA), and 22 reaches downstream from dams (hereafter “regulated”). Swedish sampling sites include large rivers with an average of the mean monthly flow of 135 m^3^/s. We selected 32 and 25 reaches in free-flowing and regulated rivers, respectively. The selection was conditioned by the existence of vegetation surveys and nearby gaging stations or, in their absence, of modeled flow data. We also ensured that reaches were well distributed along the rivers and throughout the whole study area. The combined dataset consisted of 62 free-flowing and 47 regulated sites. For Portugal, the site selection was validated to ensure that the free-flowing sites were not significantly different from the regulated in terms of geomorphology, climate and land-use (Aguiar et al., 2018). For Sweden, historic documentation indicates that prior to regulation vegetation was similar between the rivers – this has been assumed by previous studies on the same area (Nilsson et al., 1991; Nilsson and Jansson, 1995; Bejarano et al., 2018b).

In Portugal, free-flowing sites are located upstream of a dam or in a river with similar geomorphic and climatic features in relation to the respective regulated sites. Regulated reaches are mostly impaired by storage reservoirs with high productivity and smaller hydropower schemes that divert flows further downstream or directly to another reservoir. We included some run-of-river impoundments having fewer constraints in the magnitude of flows, but higher in number and duration of rise and fall rates. Rivers are mainly regulated for hydropower generation and additionally for flood defense and irrigation. The main hydrological alterations are related to a decrease of the magnitude of flows, but also to the artificial daily wetting and drying cycles (hydropeaking) and alteration of the numbers, timing and durations of seasonal floods. Regulation in Sweden included large storage reservoirs as well as run-of-river impoundments used for hydropower production through peaking operations. Therefore, regulation involved both seasonal flow stabilization resulting from the capacity of large reservoirs to store water and manage releases, and weekly and daily flow fluctuation resulting from the operation of the dams to produce hydroelectricity according to prices and demands.

## Data Collection

### Floristic and Trait Data

The floristic dataset consisted of presence/absence of woody plants (trees, shrubs, dwarf shrubs, and lianas) from Portuguese and Swedish rivers. In Portugal, surveys were carried out according to the Protocol for assessment of macrophytes and riparian woody plants in Portugal (INAG IP, 2008). Data were collected along 100-m long riparian reaches at both river margins (i.e., a total of 200 m) during late spring and early summer in 2012, 2013, and 2014 for regulated sites. The reference floristic data on free-flowing reaches were collected in 2004 and 2005 using the same protocol. The sampling area varies according to the width of the riparian zone, and averages from 1500 to 2000 m^2^. In Sweden, all woody plants between summer water level and upland forest edges were identified and noted along 200-m long riparian reaches for both free-flowing and regulated sites at one river margin during the late 1980s and early 1990s. This design was necessary to capture species at all hydrologic levels. The average sampling area was 4648 m^2^ (Jansson et al., 2000). Variation between sites can be wide, for instance, the Vindel and Ume rivers, which are known to have been very similar prior to hydroelectric development, the area of the free-flowing study sites (Vindel) varied between 1320 and 30.000 m^2^ because of geomorphic variation whereas in the regulated river (Ume) the corresponding numbers were 300 and 75.200 m^2^ (Nilsson et al., 1991). The high variation in width of the study sites in the regulated river is due to the regulation schemes. In run-of-river impoundments the riverbank is narrow because of a decrease in annual water-level fluctuations whereas in storage reservoirs with annual fluctuations of several tens of meters vertically it can be very large. In both cases, however, most of the vegetation was confined to a narrow strip close to the high-water level. The Swedish study was designed to inventory all vascular plant species. For both types of river, however, the area of the study sites would be less variable if only the area occupied by woody plants would have been measured. The combined dataset consisted of 109 species (65 in Portugal and 44 in Sweden) (Supplementary Table S1). Riparian woody communities in the Mediterranean biome overall can be discriminated with canopy height, leaf area, rooting depth, diaspore type (Stella et al., 2013; Aguiar et al., 2018), and in boreal biome, with stem flexibility, canopy height and leaf area, diaspore type, dispersal vector and reproduction type (Bejarano et al., 2018b). Trait values for each species are given in Supplementary Table S2.

### Functional Diversity Data

We used nine functional traits (from multiple organs – leaf, stem, root and reproduction characteristics) responsive to streamflow to describe the riparian vegetation (Table 2). The ecological relevance of the selected traits was obtained from Merritt et al. (2010), Nilsson et al. (2010), and Stromberg and Merritt (2016). Further information on traits’ quantitative values is given in Supplementary Table S3.

**TABLE 2:** Description of the selected functional traits used to assess functional diversity in riparian woody vegetation affected by regulation.

Trait	Definition (units)	Ecological relevance	Potential indicator
Canopy height	Shortest distance between the upper boundary of the main photosynthetic tissues on a plant and the ground level (m)	Associated with competitive vigor, whole plant fecundity and time intervals for plant growth between disturbances	Flow permanence, ground water depth
Leaf area	One-sided projected surface area of a single or an average leaf or leaf lamina (mm^2^)	Relevant for light interception, leaf energy and water balance	Water availability
Seed weight	Air dried weight of germinules or dispersules (mg)	Indicates maternal investment in individual offspring	Seedlings ability to tolerate environmental stress and inundation
Seed buoyancy	Floating capacity of diaspores on water (h)	An important role in structuring riparian communities	Plant survival and dispersal during floods
Stem flexibility	Tissue density of each species (woody and semi-woody)	Surrogate of the stem tissue density and flexibility	Hydrological variability
Rooting depth	Vertical length of the main root (deep, moderate and shallow)	Potential of an individual to acquire moisture and nutrients	Hydrological variability
Reproduction type	Type of generating new individuals (vegetatively, seeds, seeds and/or vegetatively)	One of the plant reproductive strategies	Environmental stability in riparian habitats
Diaspore type	Plant’s most common dispersal units (seeds and fruits)	Individual strategy for dispersal and establishment	Reproduction type
Dispersal vector	Transporting means of plant’s dispersal units (anemochory, hydrochory, anemochory and hydrochory; zoochory)	Facilitate continuity between spatially separated populations and determine species richness	Species’ abilities to colonize river margins

*Their definition and units, ecological relevance and potential indicators for ecosystem functioning are given.*

Trait data were gathered primarily from local databases and literature (Aguiar et al., 2013a, b; Bejarano et al., 2016), whenever this option was limited, other trait databases were used (Klotz et al., 2002; Kleyer et al., 2008).

We computed FRic and FRed indices, i.e., two metrics well adapted to presence/absence data sets as ours, to compute multidimensional trait indices (Laliberté et al., 2014). The indices were used to compute previously selected multidimensional traits. Functional Richness (FRic) reflects the range of trait diversity, i.e., how much of the functional space is occupied by different functional traits (Villéger et al., 2008). This index does not have an upper limit. Functional Redundancy (FRed) reflects the amount of saturation in multidimensional space with species with similar traits. Species are functionally redundant if they occupy the same portion of the functional space. If FRed is zero, all species are functionally different, conversely, if FRed reaches its maximum (i.e., 1) then all species are functionally identical.

### Environmental Data

We used daily streamflow from Portuguese and Swedish stations^1^ ^,2^ to compute 30 ecologically relevant hydrological attributes, which encompassed the inherent characteristics of the streamflow regime (data from Aguiar et al., 2018; Bejarano et al., 2018b). The hydrological attributes characterize the intra-annual variation in water conditions and the inter-annual changes in streamflow components before and after the alteration of the streamflow regime. They can be classified into four categories: (i) magnitude of monthly water variations, (ii) duration of annual extreme water events; (iii) frequency of high/low water pulses; (iv) rate of change in water conditions (Richter et al., 1996). Further information on the hydrological attributes is given in Supplementary Table S4.

### Data Analyses

We calculated the mean values of the selected traits. The statistical significance of the difference between the means of each functional trait, in each biome, between free-flowing and regulated sites was assessed using the Welch test or the Wilcoxon test depending on the validity of the normality assumption, which was initially checked with the Shapiro-Wilk test. Welch tests were used for the functional traits where normality is plausible, whereas the non-parametric Wilcoxon test was used for the functional traits where the normality was rejected.

We used a trait matrix (species vs traits) and a sampling matrix (sites vs species) to compute functional diversity indices. We calculated all indices in R software (R Core Team, 2014) using the R packages “FD” (Laliberté et al., 2014) and “SYNCSA” (Debastiani and Pillar, 2012). The difference in indices between countries and regulation regimes were evaluated using a two-way ANOVA with interaction (Supplementary Table S5) and the post-hoc Tukey honestly significant difference (HSD) test. The homogeneity of variances was tested with the Levene test (R package “car,” Fox and Weisberg, 2011). A 5% confidence level was considered in all tests (the null hypothesis was rejected whenever *p* < 0.05).

Two-way ANOVA was used to test the main effects and interaction of the hydrological variables with the factors “biome” (both Portugal, Sweden) and “hydrological regime” (free-flowing, regulated).

Linear models were used to analyze the relationship between the two functional diversity indices (FRic and FRed) and the hydrological attributes. Sub-model selection was carried out with an exhaustive search for the best subsets of predictors, using an efficient branch-and-bound algorithm, implemented in R package “leaps” (Lumley and Miller, 2017). Since the algorithm returns the best model of each size, the results do not depend on the choice of a cost-complexity tradeoff. Thus, it does not make any difference whether to use R^2^, adjR^2^ or AIC as the optimization criterion. The number of predictors in each sub-model was selected as follows: we accepted as plausible models those that contained less hydrological attributes but could explain over 50% of the observed variability. To ensure that the obtained sub-models were not significantly worse than the complete models (indicating the need to increase the number of predictors), we compared the best sub-models with the respective complete model using partial F-tests. As usual in multiple linear regression, given the lack of independence of residuals, the estimators of the model random errors, the adequacy of each linear model was assessed through visual inspection (Supplementary Figure S1). All analyses were performed using the R Statistical Software version 3.2.3^3^.

## Results

### Mean Functional Traits

**FIGURE 2:**
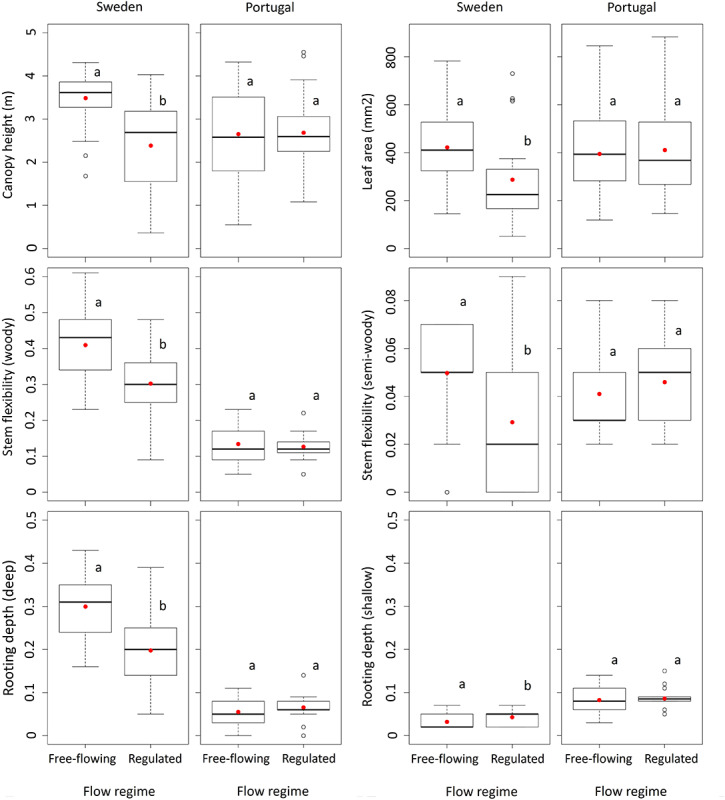
Box-and-whisker plots for canopy height, leaf area, stem flexibility and rooting depth from free-flowing and regulated sites in Sweden and Portugal. Letters identify the significantly different trait values (*p* < 0.05). Red circles represent trait averages. Canopy height and leaf area represent continuous traits, stem flexibility and rooting depth represent categorical traits. For assessment of all traits (*n* = 9), please consult Supplementary Figure S2.

We observed significant differences in average characteristics of plant species between free-flowing and regulated sites in Sweden for canopy height, leaf area, seed weight, rooting depth, stem flexibility, diaspore type and dispersal vector. There were no differences in seed buoyancy and reproduction from seeds. We also found that regulation enabled riparian woody species with small size, tiny leaves, poorly lignified stems, and shallow roots to persist at regulated sites in Sweden (Figure 2). In Portugal, the average trait values of plant species were not significantly different between free-flowing and regulated sites (Figure 2, Supplementary Figure S2).

### Functional Diversity Indices

**FIGURE 3:**
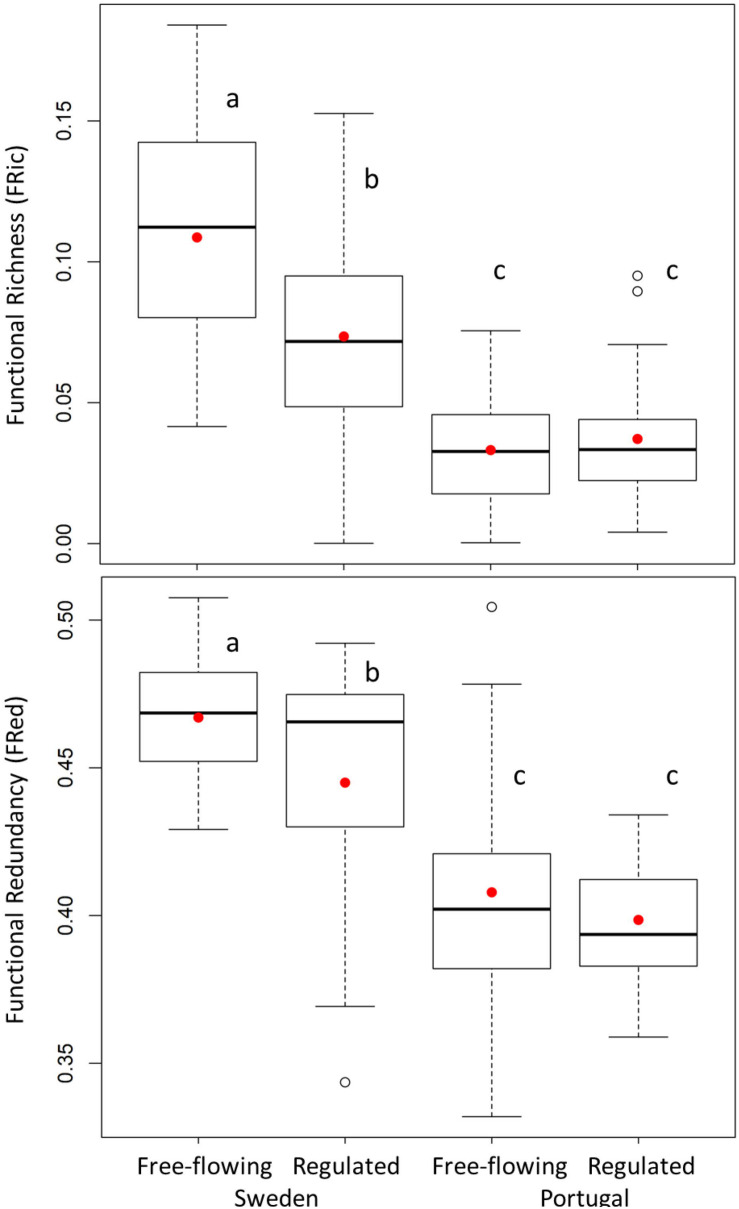
Functional diversity indices (Functional Richness and Functional Redundancy) from free-flowing and regulated sites in Sweden and Portugal. Letters identify significantly different means (red circles) (*p* < 0.05).

We observed a significant decrease in FRic and FRed with regulation in Sweden and a non-significant variation in Portugal (Figure 3 and Supplementary Figure S3). This result is supported by the previous functional trait analyses, showing that filtering out of certain traits leads to constrained FRic and redundancy in Sweden. On the contrary, in Portugal, due to the persistence of the same functional traits in regulated sites, functional diversity indices did not differ significantly between free-flowing and regulated sites (Supplementary Table S6).

### Hydrological Attributes

The streamflow regulation had distinct impacts on the hydrological attributes between the biomes. Some similarities in regulation between biomes were observed (such as maximum flows in summer months and high pulse count). However, the magnitude of the hydrological alterations and the number of hydrological variables affected was smaller in Portugal than in Sweden (Supplementary Tables S7, S8).

Two-way ANOVA revealed that the regulation significantly decreased FRic and FRed in Sweden but not in Portugal (Figure 3). An explanation of the effect of river regulation on functional diversity was only searched for in Sweden, since for Portugal the effect of regulation was not significant. The results of multiple linear regressions, with each functional diversity index as a response variable and a set of hydrological attributes, optimally chosen, as predictors, are presented in Table 3.

**TABLE 3:** Coefficients of the hydrological attributes used in the linear submodels for FRic and FRed for Swedish rivers, considering FRic (FRed) = β_0_ + ∑_*i*_β_*i*_*x*_*i*_ + ε where *x*_*i*_ represents an hydrological attribute (predictor) and ε is the random error, supposed normally distributed with zero mean.

Category	Hydrological attribute	Functional richness	*P*-value	Functional redundancy	*P*-value
Magnitude of monthly water variations	June mean flow	–0.003	0.04		
	August mean flow	0.001	< 0.001		
Duration of annual extreme water events	1-day minimum			0.003	< 0.001
	7-day minimum			–0.004	< 0.001
	1-day maximum	0.005	< 0.001		
	7-day maximum			–0.001	0.0001
	90-day maximum	–0.001	0.0002		
Frequencies of high/low water pulses	High pulse count			–0.03	< 0.001
Rate of change	Rise rate			0.008	< 0.001
	No. of hydrologic reversals	–0.001	< 0.001	–0.001	0.0002
R^2^		0.50		0.65	
AdjR^2^		0.45		0.60	
*P*-value		< 0.001		< 0.001	

*FRic (FRed) uses 5 (6) predictors. R^2^, AdjR^2^ and *P*-value of global *F*-test of the selected models are given. Global *F*-test tests the hypothesis H0: all β_*i*_ = 0 (null model) vs H1: at least one β_*i*_ = 0.*

In general, the hydrological attributes in the categories “Duration of annual extreme water events” and “Rate of changes” were key for both FRic and FRed in Swedish rivers. “Magnitude of monthly water variations” was an additional descriptor for FRic and “Frequent pulses of high water” for FRed. The best model for FRic explained 65% of the total variability and consisted of five streamflow attributes. Mean June streamflow, 90-day moving average of maximum streamflow and number of daily reversals had negative effects, whereas mean streamflow in August and 1-day moving average maximum had positive effects on FRic values. The best model for FRed explained 51% of the total variability: 7-day moving averages of minimum and maximum flows and frequencies of high pulses and daily streamflow reversals had negative effects on FRed, whereas the 1-day moving average of minimum streamflow and rise rates affected FRed positively.

## Discussion

In line with the assumptions, our results showed that the effect of regulation can differ among biomes, likely related to species’ natural adaptations to flow-related disturbances and to the magnitude of the hydrological alterations. In the boreal region, the changed streamflow regime disfavored certain traits that shaped the riparian woody vegetation in free-flowing sites, leading to a reduced range of traits in regulated sites.

### Regulation Effect on Functional Traits

In regulated rivers of the boreal region, we observed species with lower canopies, smaller leaves, and more flexible stems, all being disturbance-tolerant traits typically linked to high-flow-velocity environments. More compact plants are more resistant to mechanical disturbance from flowing water and flexible stems reduce the risk of biomass loss because of fast flows (Madsen et al., 2001). Rooting depth, which is considered as a stress indicator of water availability and which may be extensive in dry soils, was lower in regulated rivers. This may be a result of the almost constantly moist riverbanks following flow releases from upstream reservoirs under which conditions riparian vegetation does not need to invest in root elongation for water uptake (West et al., 2012). Therefore, given the climate in Sweden, even during water recession, the risk of water stress is limited, suggesting that traits resistant to water stress may be irrelevant. Although vegetative propagation has been reported in rivers subjected to high or low fluvial disturbances (Bellingham and Sparrow, 2000; Riis and Sand-Jensen, 2006) and in relatively stable riparian conditions (Douhovnikoff et al., 2005), in our case, species with seed regeneration persisted. It was noticeable, however, that heavy seeds were disfavored by regulation, most probably because the repeated flood events may facilitate the transport of light seeds downstream of dams, where plant establishment will be more likely (Johansson et al., 1996). The concentration of transported seeds remains unknown, because even if floods can assist in seed transport, their concentration may be drastically reduced due to the difficulties for plants to pass dams (Merritt and Wohl, 2006).

In Mediterranean regions, riparian communities of woody species in regulated rivers may be affected by suppressed stream flows and largely variable flood patterns (Magdaleno and Fernández, 2011) which are comparable to natural hydrological regimes, and may result in some common functionality patterns (Belmar et al., 2019). In fact, the observed small streamflow changes with regulation in Portugal did not change the already existing pool of traits, with trait values remaining similar between free-flowing and regulated sites. Further, there is evidence that traits in free-flowing rivers of Mediterranean-climate regions may occur also in regulated ones as an adaptation to natural hydrological stress (Stella et al., 2013). In accordance, the observed short plants can be a result of water shortage, i.e., less time to grow to maturity (Pakeman and Eastwood, 2013); semi-woody characteristics and large leaves are adaptations to rapid growth during periods of water supply (Grady et al., 2013; Lawson et al., 2015a); and persistence of deep roots an adaptation to fluctuating water levels (Schenk and Jackson, 2002).

### Regulation Effect on Functional Diversity Indices

It is showed that dams alter streamflow across biogeographic regions (Poff and Zimmerman, 2010), but the alterations depend not only on dam operation but also on the regional hydrological context (McManamay et al., 2012). In that sense, regulation of large rivers as in those of the boreal region may have severe consequences for riparian vegetation (Nilsson et al., 2005). Several reasons make regulation stand out as a strong factor impacting riparian woody vegetation in the boreal region. First, upstream impoundments and canals downstream of dams used for hydropower production are subjected to hydropeaking, which involves high within-day and day-to-day variations in flow and water-level. Second, storage reservoirs have large water-level magnitudes and a reversed flow regime. In both these cases of regulation, the environment may be harsher than plants can tolerate and consequently result in species loss since only a few species share traits adequate for such novel hydrology (Catford and Jansson, 2014). The strong filtering effect decreases trait space occupied by communities and limits functional overlap (Bruno et al., 2016; de la Riva et al., 2017). The absence of compensatory dynamics in communities with limited functional richness and redundancy decreases the capacity of species to buffer disturbances (Elmqvist et al., 2003; Pillar et al., 2013).

Regulation of rivers in the Mediterranean region does not seem to impose further stress to similar extents as regulated rivers in the boreal region. Indeed, there was no significant change in FRic and FRed and only constrained variability in functional diversity. This is likely due to two reasons. First, hydrological stress is typical for Mediterranean regions under natural, free-flowing conditions, and over evolutionary scales it has shaped communities by exposing them to rapid shifts between droughts and floods (Bonada and Resh, 2013). Second, the streamflow alteration induced by regulation was not markedly distinct from the natural streamflow. Those combined exposures could explain why regulation in the Mediterranean region did not cause any significant trait loss in the resident plant communities and consequently, did not result in a reduction of FRic and redundancy. A similar observation was made by Aguiar et al. (2018) who found that, for rivers in Mediterranean Europe, riparian woody communities did not change their trait composition following river regulation, but several trait values became less abundant. According to Sandel et al. (2010), such reductions of trait abundances may precede functional diversity loss.

### Effect of Regulated Streamflow Attributes on Functional Diversity

We found that the rate of change, frequency of high pulses, duration of extreme water events and monthly mean streamflow all had a significant impact on the functional diversity of boreal rivers. The reduced functional diversity due to the increased daily streamflow changes can be related to scouring capacity of moving water, resulting in mechanical damage or riparian plant removal (Bejarano et al., 2018a). Further negative effect on functional diversity was imposed by the frequent high pulses and prolonged duration of extreme high-water events (90-day moving average maximum and 7-day moving average maximum). Under an extended duration of inundation in riparian areas, physiological processes are hampered, consequently reducing the survival and growth of riparian vegetation (Johansson and Nilsson, 2002). Similarly, an extended duration of extreme low-water conditions (7-day moving minimum) can reduce functional diversity due to soil moisture deficits. While the prolonged duration of both inundation and low-water conditions cause negative effect on riparian vegetation, 1-day hydrological events are too short to cause severe disturbance. In fact, they may even support the transport of propagules and nutrients and remove or create new habitats for plant establishment (Corenblit et al., 2007), resulting in increased functional diversity. However, the positive effect of rise rates on riparian vegetation was surprising nevertheless, in natural streamflow regimes, rapid rise rates have also been linked to functional heterogeneity (Lawson et al., 2015b). We also observed that timing of the monthly streamflow can have consequences on riparian vegetation. For instance, the June mean streamflow overlaps with the boreal growing season, which typically occurs between May and October. Thus, the combined effect of the natural early summer flood and higher June mean streamflow may reduce germination due to the long period of waterlogged soils (Sarneel et al., 2019). Such a condition may disrupt plant establishment and reduce functional diversity since most plant species have lower flood tolerance during the growing season (Siebel and Blom, 1998).

## Limitations

We showed that the functional diversity approach can be used to evaluate the impacts of streamflow regulation on riparian woody communities. However, some methodological aspects deserve further explanation. First, the chosen functional diversity indices. The available dataset of species presence/absence permits the use of indices computed with binary data, namely FRic and FRed. Articles on functional diversity consisting of binary data sets have been published on riparian vegetation (Sonnier et al., 2014; Brice et al., 2017) and those datasets have been considered as reliable for predicting plant trait distributions globally (Boonman et al., 2020). In a conceptual study with an illustrated ecological hypothesis, Boersma et al. (2016) stated that presence/absence data can serve to make the most straightforward interpretation of the results when disturbance acts as an environmental filter. Nevertheless, possible bias may rise when the filtering factor does not have a significant effect on communities, for instance, due to intrinsic adaptations. Therefore, the effect might not be projected in species loss but rather in abundance change. Under that assumption, species abundance likely can be important for the Mediterranean riparian woody communities, as species are resilient to disturbance, and still occur in the riparian zone (Aguiar et al., 2018). Second, selection and collection of traits (Lozanovska et al., 2018a). In this regard, the selection of many functional traits increases the ability to detect functional differences between species, thereby increasing the estimate of functional diversity (Fonseca and Ganade, 2001). On the other hand, considering few traits may undervalue functional diversity (Petchey and Gaston, 2002). Therefore, to allow for deeper insights in functional diversity–ecosystem functioning relationships, the number of traits should be balanced and measured from multiple organs such as leaves, stems and roots (Laughlin, 2014). In the present study, we selected nine functional traits that summarize adaptations of riparian woody vegetation to deal with anoxia, drought and fluvial disturbances. However, using a “performance trait” which contributes directly to fitness (i.e., ability of a species to grow, reproduce or survive) instead of “functional trait” which has an impact on performance traits and thus indirectly on fitness may provide more accurate indications of functional diversity and ecosystem functioning (Violle et al., 2007). Third, the temporal and spatial aspects of the study. Due to the differences in the timing of the data collection in Portugal and Sweden, a time lag might affect the results. Although the effect potentially may decrease the observation of functional differences between biomes, we have assumed that the influence would be smaller compared to the effect of regulation on riparian woody communities. The difference in sampling areas reflects the smaller riparian zones in Portugal compared to the larger ones in Sweden. Extending the sampling area in Portugal to be equal to Sweden, would mean the inclusion of species from the terrestrial zone. Nevertheless, the fact that the species number in Portugal is higher despite the smaller sampling area strongly suggests that differences in species richness are not a sampling area effect.

## Conclusion

Previous studies have recognized that altered streamflow regimes can lead to shifts, and loss of traits and species (Kominoski et al., 2013), and ultimately loss of ecosystem functions, thus jeopardizing ecosystem services provided by riparian ecosystems (Cadotte et al., 2011). We showed that the differences induced by regulation between the Mediterranean and boreal riparian woody vegetation are related to species legacy adaptations, and differences in the magnitude of streamflow alteration. Also, streamflow regulation can foster stress-related functional strategies to deal with regulation, resulting in functional diversity reduction.

Our study highlights the potential of functional measures for monitoring riparian vegetation changes caused by dam-induced hydrological alterations. By specifically targeting and managing the streamflow attributes, functional diversity may potentially be maintained or even improved. To facilitate such actions, future research should focus on the tolerance limits of species or certain functional traits to specific hydrological variables that are altered as a result of river regulation.

## Data Availability Statement

The datasets generated for this study are available on request to the corresponding author.

## Author Contributions

All authors conceived the ideas, discussed and interpreted results, contributed critically to the manuscript’s drafts and revised them for important intellectual content, and gave final approval for publication. MM, FA, and IL designed the methodology and analyzed the data. IL, FA, and MM led the writing.

## Conflict of Interest

The authors declare that the research was conducted in the absence of any commercial or financial relationships that could be construed as a potential conflict of interest.

## Footnotes

1 https://snirh.apambiente.pt/

2 http://vattenwebb.smhi.se/

3 www.r-project.org
