# Supplementary material for: Functional Diversity of Riparian Woody Vegetation Is Less Affected by River Regulation in the Mediterranean Than Boreal Region
Source: Front Plant Sci. 2020 Jun 24;11:857. doi: 10.3389/fpls.2020.00857 (PMC7327385; doi:10.3389/fpls.2020.00857)
Supplement: Supplementary file 1 [file Data_sheet_1.docx]

**Supplementary material**

**Functional diversity of riparian woody vegetation is less affected by river regulation in the Mediterranean than boreal region**

Ivana Lozanovska^a*^, María Dolores Bejarano^b^, Maria João Martins^a^, Christer Nilsson^b,c^, Maria Teresa Ferreira^a^, Francisca C. Aguiar^a^

Corresponding author: ivana.lozanovska@gmail.com

**Supplementary Table S1.** Species list, family, origin and respective country.

| **Species** | **Family** | **Origin** | **Country** |
| --- | --- | --- | --- |
| *Acacia dealbata* Link | FABACEAE | Exotic | Portugal |
| *Acacia melanoxylon* R. Br. | FABACEAE | Exotic | Portugal |
| *Acer pseudoplatanus* L. | ACERACEAE | Native | Portugal |
| *Ailanthus altissima* (Miller) Swingle | SIMAROUBACEAE | Exotic | Portugal |
| *Alnus glutinosa* (L.) Gaertner | BETULACEAE | Native | Portugal |
| *Alnus incana* (L.) Moench | BETULACEAE | Native | Sweden |
| *Andromeda polifolia* L. | ERICACEAE | Native | Sweden |
| *Arbutus unedo* L. | ERICACEAE | Native | Portugal |
| *Arctostaphylos alpinus* (L.) Sprengel | ERICACEAE | Native | Sweden |
| *Arctostaphylos uva-ursi* (L.) Sprengel | ERICACEAE | Native | Sweden |
| *Betula nana* L. | BETULACEAE | Native | Sweden |
| *Betula pendula* Roth | BETULACEAE | Native | Sweden |
| *Betula pubescens* Ehrh. | BETULACEAE | Native | Sweden |
| *Betula pubescens* Ehrh. subsp. *celtiberica* (Rothm. & Vasc) Rivas Martin. | BETULACEAE | Native | Portugal |
| *Calluna vulgaris* (L.) Hull | ERICACEAE | Native | Portugal; Sweden |
| *Castanea sativa* Miller | FAGACEAE | Exotic | Portugal |
| *Cistus psilosepalus* Sweet | CISTACEAE | Native | Portugal |
| *Corylus avellana* L. | BETULACEAE | Native | Portugal |
| *Crategus monogyna* Jacq. | ROSACEAE | Native | Portugal |
| *Cytisus scoparius* (L.) Link | FABACEAE | Native | Portugal |
| *Cytisus striatus* (Hill.) Rothm | FABACEAE | Native | Portugal |
| *Daphne gnidium* L. | THYMELAEACEAE | Native | Portugal |
| *Daphne mezereum* L. | THYMELAEACEAE | Native | Sweden |
| *Dittrichia viscosa* (L.) W. Greuter | ASTERACEAE | Native | Portugal |
| *Empetrum nigrum* L. subsp*. hermaphroditum* (Lange ex Hagerup) Böcher | ERICACEAE | Native | Sweden |
| *Erica arborea* L. | ERICACEAE | Native | Portugal |
| *Erica australis* L. | ERICACEAE | Native | Portugal |
| *Erica ciliaris* L. | ERICACEAE | Native | Portugal |
| *Erica cinerea* L. | ERICACEAE | Native | Portugal |
| *Erica tetralix* L. | ERICACEAE | Native | Portugal |
| *Ficus carica* L. | MORACEAE | Exotic | Portugal |
| *Flueggea tinctoria* (L.) G.L. Webster | PHYLLANTHACEAE | Native | Portugal |
| *Frangula alnus* Mill. | RHAMNACEAE | Native | Portugal; Sweden |
| *Fraxinus angustifolia* Vahl | OLEACEAE | Native | Portugal |
| *Genista florida* L. | FABACEAE | Native | Portugal |
| *Hedera hibernica* (G. Kirchn.) Bean | ARALIACEAE | Native | Portugal |
| *Hypericum androsaemum* L. | CLUSIACEAE | Native | Portugal |
| *Hypericum humifussum* L. | CLUSIACEAE | Native | Portugal |
| *Ilex aquifolium* L*.* | AQUIFOLIACEAE | Native | Portugal |
| *Juglans regia* L. | JUGLANDACEAE | Exotic | Portugal |
| *Juniperus communis* L. | CUPRESSACEAE | Native | Sweden |
| *Laurus nobilis* L. | LAURACEAE | Native | Portugal |
| *Lavandula stoechas* L. | LAMIACEAE | Native | Portugal |
| *Ledum palustre* L. | ERICACEAE | Native | Sweden |
| *Linnaea borealis* L. | CAPRIFOLIACEAE | Native | Sweden |
| *Lithodora prostrata* (Loisel.) Griseb | BORAGINACEAE | Native | Portugal |
| *Lycopodium annotinum* L. | LYCOPODIACEAE | Native | Sweden |
| *Lycopodium clavatum* L. | LYCOPODIACEAE | Native | Sweden |
| *Lycopodium selago* L*.* (*Huperzia selago* (L.) Bernh. Ex Schrank & Mart.) | LYCOPODIACEAE | Native | Sweden |
| *Myrica gale* L. | MYRICACEAE | Native | Sweden |
| *Myrtus communis* L. | MYRTACEAE | Native | Portugal |
| *Phillyrea angustifolia* L. | OLEACEAE | Native | Portugal |
| *Phillyrea latifolia* L. | OLEACEAE | Native | Portugal |
| *Picea abies* (L.) H.Karst. | PINACEAE | Native | Sweden |
| *Pinus pinaster* Aiton | PINACEAE | Native | Portugal |
| *Pinus sylvestris* L. | PINACEAE | Native | Sweden |
| *Platanus hispanica* Miller | PLANTAGINACEAE | Exotic | Portugal |
| *Populus x canadensis* Moench | SALICACEAE | Exotic | Portugal |
| *Populus tremula* L. | SALICACEAE | Native | Sweden |
| *Prunus avium* L. | ROSACEAE | Native | Portugal |
| *Prunus padus* L. | ROSACEAE | Native | Sweden |
| *Pyrus cordata* Desv. | ROSACEAE | Native | Portugal |
| *Quercus faginea* Lam. subsp. *broteroi* (Coutinho) A. Camus | FAGACEAE | Native | Portugal |
| *Quercus pyrenaica* Willd. | FAGACEAE | Native | Portugal |
| *Quercus robur* L. | FAGACEAE | Native | Portugal |
| *Quercus suber* L. | FAGACEAE | Native | Portugal |
| *Ribes nigrum* L*.* | GROSSULARIACEAE | Native | Sweden |
| *Ribes rubrum* L*.* | GROSSULARIACEAE | Native | Sweden |
| *Rosa canina* L. | ROSACEAE | Native | Portugal |
| *Rosa majalis* J. Herrmann | ROSACEAE | Native | Sweden |
| *Rosa pouzinii* Tratt. | ROSACEAE | Native | Portugal |
| *Rubus brigantinus* Samp. | ROSACEAE | Native | Portugal |
| *Rubus genevieri* Boreau | ROSACEAE | Native | Portugal |
| *Rubus idaeus* L. | ROSACEAE | Native | Sweden |
| *Rubus lainzii* H. E. Weber | ROSACEAE | Native | Portugal |
| *Rubus radula* Weihe | ROSACEAE | Native | Portugal |
| *Rubus ulmifolius* Schott | ROSACEAE | Native | Portugal |
| *Rubus vagabundus* Samp. | ROSACEAE | Native | Portugal |
| *Salix alba* L. | SALICACEAE | Native | Portugal |
| *Salix atrocinerea* Brot. | SALICACEAE | Native | Portugal |
| *Salix aurita* L. | SALICACEAE | Native | Sweden |
| *Salix caprea* L. | SALICACEAE | Native | Sweden |
| *Salix cinerea* L. | SALICACEAE | Native | Sweden |
| *Salix glauca* L. | SALICACEAE | Native | Sweden |
| *Salix hastata* L*.* | SALICACEAE | Native | Sweden |
| *Salix herbacea* L. | SALICACEAE | Native | Sweden |
| *Salix lanata* L*.* | SALICACEAE | Native | Sweden |
| *Salix lapponum* L*.* | SALICACEAE | Native | Sweden |
| *Salix myrsinites* L. | SALICACEAE | Native | Sweden |
| *Salix myrtilloides* L. | SALICACEAE | Native | Sweden |
| *Salix neotricha* Goerz | SALICACEAE | Native | Portugal |
| *Salix pentandra* L*.* | SALICACEAE | Native | Sweden |
| *Salix salviifolia* Brot. | SALICACEAE | Native | Portugal |
| *Salix triandra* L. | SALICACEAE | Native | Sweden |
| *Sambucus nigra* L. | CAPRIFOLIACEAE | Native | Portugal |
| *Sesamoides suffruticosa* (Lange) Kuntze | RESEDACEAE | Native | Portugal |
| *Sorbus aucuparia* L. | ROSACEAE | Native | Portugal; Sweden |
| *Thymus mastichina* (L.) L. | LAMIACEAE | Native | Portugal |
| *Ulex minor* Roth | FABACEAE | Native | Portugal |
| *Vaccinium microcarpum* (Turcz. ex Rupr.) Schmalh. | ERICACEAE | Native | Sweden |
| *Vaccinium myrtillus* L. | ERICACEAE | Native | Portugal; Sweden |
| *Vaccinium oxycoccos* L. | ERICACEAE | Native | Sweden |
| *Vaccinium uliginosum* L. | ERICACEAE | Native | Sweden |
| *Vaccinium vitis-idaea* L. | ERICACEAE | Native | Sweden |
| *Vitis vinifera* L. subsp. *vinifera* | VITACEAE | Native | Portugal |

**Supplementary Table S2.** Trait values for the riparian woody species assessment in countries Portugal and Sweden.

| **Species** | **Canopy height (m)** | **Leaf area (mm^2^)** | **Seed buoyancy (h)** | **Seed weight (g)** | **Stem flexibility** | **Rooting depth** | **Reproduction type** | **Diaspore type** | **Dispersal vector** |
| --- | --- | --- | --- | --- | --- | --- | --- | --- | --- |
| *Acacia dealbata* | 30 | 3300 | 400 | 12.7 | Woody | Moderate | Seeds and/or vegetatively | Fruits | Anemochory, hydrochory, anemochory+hydrochory |
| *Acacia melanoxylon* | 30 | 2100 | 400 | 14 | Woody | Moderate | Seeds and/or vegetatively | Seeds | Anemochory, hydrochory, anemochory+hydrochory |
| *Acer pseudoplatanus* | 25 | 8332 | 168 | 94.4 | Woody | Deep | Seeds | Fruits | Anemochory, hydrochory, anemochory+hydrochory |
| *Ailanthus altissima* | 30 | 1155 | 480 | 29.4 | Woody | Shallow | Seeds and/or vegetatively | Fruits | Anemochory, hydrochory, anemochory+hydrochory |
| *Alnus glutinosa* | 25 | 2735 | 8760 | 4 | Woody | Deep | Seeds | Seeds | Anemochory, hydrochory, anemochory+hydrochory |
| *Alnus incana* | 14 | 2734.08 | 2544 | 1.4 | Woody | Shallow | Seeds and/or vegetatively | Fruits | Anemochory, hydrochory, anemochory+hydrochory |
| *Andromeda polifolia* | 0.2 | 80.75 | 72 | 0.2 | Woody | Shallow | Vegetativley | Seeds | Anemochory, hydrochory, anemochory+hydrochory |
| *Arbutus unedo* | 7 | 1038 | 0.02 | 5.7 | Woody | Shallow | Seeds and/or vegetatively | Fruits | Zoochory |
| *Arctostaphylos alpinus* | 0.2 | 137.93 | 240 | 2.6 | Woody | Shallow | Seeds and/or vegetatively | Fruits | Zoochory |
| *Arctostaphylos*  *uva-ursi* | 0.1 | 113 | 240 | 18.5 | Woody | Shallow | Seeds and/or vegetatively | Fruits | Zoochory |
| *Betula nana* | 0.5 | 48.24 | 0 | 0.38 | Woody | Moderate | Vegetativley | Fruits | Anemochory, hydrochory, anemochory+hydrochory |
| *Betula pendula* | 16.5 | 1000 | 5640 | 0.5 | Woody | Deep | Seeds | Fruits | Anemochory, hydrochory, anemochory+hydrochory |
| *Betula pubescens* | 14 | 1000 | 2184 | 0.76 | Woody | Deep | Seeds | Fruits | Anemochory, hydrochory, anemochory+hydrochory |
| *Betula pubescens* subsp*. celtiberica* | 20 | 1000 | 168 | 0.12 | Woody | Moderate | Seeds | Fruits | Zoochory |
| *Calluna vulgaris* | 2 | 1 | 96 | 0.03 | Woody | Shallow | Vegetativley | Seeds | Anemochory, hydrochory, anemochory+hydrochory |
| *Calluna vulgaris* | 0.3 | 8 | 96 | 0.03 | Woody | Shallow | Vegetativley | Seeds | Anemochory, hydrochory, anemochory+hydrochory |
| *Castanea sativa* | 30 | 7898 | 24 | 9944 | Woody | Deep | Seeds | Fruits | Zoochory |
| *Cistus psilosepalus* | 1.2 | 339 | 48 | 0.92 | Semi-woody | Shallow | Seeds | Seeds | Zoochory |
| *Corylus avellana* | 8 | 4024 | 420 | 1097.3 | Woody | Deep | Seeds and/or vegetatively | Fruits | Zoochory |
| *Crataegus monogyna* | 10 | 610 | 168 | 98 | Woody | Deep | Seeds and/or vegetatively | Fruits | Zoochory |
| *Cytisus scoparius* | 2 | 22.4 | 336 | 9 | Woody | Deep | Seeds | Seeds | Anemochory, hydrochory, anemochory+hydrochory |
| *Cytisus striatus* | 3 | 25 | 336 | 7.8 | Woody | Deep | Seeds | Seeds | Anemochory, hydrochory, anemochory+hydrochory |
| *Daphne gnidium* | 2 | 125 | 336 | 7.61 | Woody | Deep | Seeds and/or vegetatively | Fruits | Zoochory |
| *Daphne mezereum* | 0.75 | 930 | 336 | 85.9 | Woody | Moderate | Seeds | Fruits | Zoochory |
| *Dittrichia viscosa* | 1.3 | 157.5 | 60 | 0.3638 | Semi-woody | Shallow | Seeds | Fruits | Zoochory |
| *Empetrum nigrum* subsp. *hermaphroditum* | 0.2 | 3.14 | 408 | 1.03 | Woody | Shallow | Seeds and/or vegetatively | Fruits | Zoochory |
| *Erica arborea* | 7 | 3.6 | 480 | 0.2 | Woody | Moderate | Seeds and/or vegetatively | Seeds | Zoochory |
| *Erica australis* | 2.5 | 3 | 168 | 0.2172 | Woody | Moderate | Seeds and/or vegetatively | Seeds | Zoochory |
| *Erica ciliaris* | 1.8 | 3.3 | 168 | 0.04 | Woody | Moderate | Seeds and/or vegetatively | Seeds | Zoochory |
| *Erica cinerea* | 0.75 | 2.2 | 168 | 0.13 | Woody | Moderate | Seeds and/or vegetatively | Seeds | Zoochory |
| *Erica tetralix* | 0.7 | 3.7 | 168 | 0.02 | Woody | Moderate | Seeds and/or vegetatively | Seeds | Zoochory |
| *Ficus carica* | 10 | 18787 | 0.01 | 0.1 | Woody | Moderate | Seeds and/or vegetatively | Fruits | Zoochory |
| *Flueggea tinctoria* | 2 | 0.84 | 168 | 0.004 | Woody | Moderate | Seeds and/or vegetatively | Seeds | Zoochory |
| *Frangula alnus* | 4 | 1410 | 360 | 20.6 | Woody | Shallow | Seeds and/or vegetatively | Fruits | Zoochory |
| *Frangula alnus* | 5 | 961 | 360 | 20.6 | Woody | Shallow | Seeds and/or vegetatively | Fruits | Zoochory |
| *Fraxinus angustifolia* | 25 | 382 | 6 | 49.4 | Woody | Deep | Seeds | Fruits | Anemochory, hydrochory, anemochory+hydrochory |
| *Genista florida* | 2 | 364 | 168 | 6.63 | Woody | Shallow | Seeds | Seeds | Zoochory |
| *Hedera hibernica* | 30 | 4852 | 168 | 16.85 | Woody | Shallow | Seeds and/or vegetatively | Fruits | Anemochory, hydrochory, anemochory+hydrochory |
| *Hypericum androsaemum* | 2 | 404 | 168 | 0.07 | Semi-woody | Shallow | Seeds | Seeds | Zoochory |
| *Hypericum humifussum* | 0.3 | 35 | 168 | 0.0368 | Semi-woody | Shallow | Seeds | Seeds | Zoochory |
| *Ilex aquifolium* | 12 | 1608 | 240 | 36.1 | Woody | Moderate | Seeds and/or vegetatively | Fruits | Zoochory |
| *Juglans regia* | 25 | 3909 | 6 | 11490 | Woody | Deep | Seeds | Fruits | Zoochory |
| *Juniperus communis* | 3.2 | 20.4 | 528 | 12.5 | Woody | Moderate | Seeds and/or vegetatively | Fruits | Anemochory, hydrochory, anemochory+hydrochory |
| *Laurus nobilis* | 10 | 3675 | 168 | 0.19 | Woody | Deep | Seeds | Fruits | Anemochory, hydrochory, anemochory+hydrochory |
| *Lavandula stoechas* | 0.87 | 27 | 168 | 0.9 | Semi-woody | Shallow | Seeds | Seeds | Zoochory |
| *Ledum palustre* | 1.5 | 206.5 | 168 | 0.006 | Woody | Moderate | Seeds and/or vegetatively | Seeds | Anemochory, hydrochory, anemochory+hydrochory |
| *Linnaea borealis* | 0.1 | 76 | 12 | 2 | Semi-woody | Shallow | Vegetativley | Fruits | Zoochory |
| *Lithodora prostrata* | 60 | 900 | 60 | 8.6 | Semi-woody | Shallow | Seeds | Seeds | Zoochory |
| *Lycopodium annotinum* | 0.3 | 5.8 | 0 | 0 | Semi-woody | Shallow | Seeds | Seeds | Zoochory |
| *Lycopodium clavatum* | 0.16 | 2.3 | 0 | 0 | Semi-woody | Shallow | Seeds | Seeds | Anemochory, hydrochory, anemochory+hydrochory |
| *Lycopodium selago* | 0.15 | 3.9 | 0 | 0 | Semi-woody | Shallow | Seeds | Seeds | Anemochory, hydrochory, anemochory+hydrochory |
| *Myrica gale* | 1.05 | 188 | 8760 | 1.57 | Woody | Shallow | Seeds and/or vegetatively | Fruits | Anemochory, hydrochory, anemochory+hydrochory |
| *Myrtus communis* | 5 | 325 | 14 | 8.76 | Woody | Deep | Seeds and/or vegetatively | Fruits | Zoochory |
| *Phillyrea angustifolia* | 4 | 278 | 14 | 36 | Woody | Shallow | Seeds and/or vegetatively | Fruits | Zoochory |
| *Phillyrea latifolia* | 8 | 419 | 14 | 19 | Woody | Shallow | Seeds and/or vegetatively | Fruits | Zoochory |
| *Picea abies* | 40 | 39.8 | 360 | 7 | Woody | Shallow | Seeds and/or vegetatively | Seeds | Anemochory, hydrochory, anemochory+hydrochory |
| *Pinus pinaster* | 40 | 250 | 6 | 47 | Woody | Deep | Seeds | Seeds | Anemochory, hydrochory, anemochory+hydrochory |
| *Pinus sylvestris* | 30 | 87.3 | 360 | 6 | Woody | Shallow | Seeds | Seeds | Anemochory, hydrochory, anemochory+hydrochory |
| *Platanus hispanica* | 35 | 3570 | 168 | 3 | Woody | Deep | Seeds | Seeds | Anemochory, hydrochory, anemochory+hydrochory |
| *Populus tremula* | 20 | 2556.3 | 168 | 0.12 | Woody | Shallow | Seeds and/or vegetatively | Seeds | Anemochory, hydrochory, anemochory+hydrochory |
| *Populus x canadensis* | 40 | 6250 | 168 | 0.9 | Woody | Deep | Seeds | Seeds | Zoochory |
| *Prunus avium* | 30 | 5775 | 24 | 186 | Woody | Moderate | Seeds | Fruits | Zoochory |
| *Prunus padus* | 7 | 2978 | 12 | 84 | Woody | Moderate | Seeds and/or vegetatively | Fruits | Zoochory |
| *Pyrus cordata* | 15 | 1408 | 24 | 9.98 | Woody | Moderate | Seeds | Fruits | Zoochory |
| *Quercus faginea* subsp. *broteroi* | 20 | 1018 | 168 | 2510 | Woody | Deep | Seeds and/or vegetatively | Fruits | Zoochory |
| *Quercus pyrenaica* | 25 | 9600 | 168 | 155.4 | Woody | Moderate | Seeds | Fruits | Zoochory |
| *Quercus robur* | 40 | 3281 | 168 | 3378 | Woody | Deep | Seeds | Seeds | Zoochory |
| *Quercus suber* | 25 | 2406.25 | 168 | 3469 | Woody | Deep | Seeds and/or vegetatively | Fruits | Zoochory |
| *Ribes nigrum* | 1.25 | 5979 | 0.33 | 0.7 | Woody | Deep | Seeds | Fruits | Anemochory, hydrochory, anemochory+hydrochory |
| *Ribes rubrum* | 1.4 | 6172 | 0.33 | 4.1 | Woody | Shallow | Seeds | Fruits | Zoochory |
| *Robinia pseudoacacia* | 25 | 962 | 200 | 19.2 | Woody | Deep | Seeds and/or vegetatively | Fruits | Anemochory, hydrochory, anemochory+hydrochory |
| *Rosa canina* | 6 | 296 | 24 | 16 | Semi-woody | Shallow | Seeds | Fruits | Zoochory |
| *Rosa majalis* | 1.1 | 703.1 | 30 | 0.04 | Woody | Shallow | Seeds and/or vegetatively | Fruits | Zoochory |
| *Rosa pouzinii* | 3 | 750 | 36 | 11.17 | Semi-woody | Shallow | Seeds | Fruits | Zoochory |
| *Rubus brigantinus* | 2 | 1350 | 14 | 2.58 | Semi-woody | Shallow | Seeds and/or vegetatively | Fruits | Anemochory, hydrochory, anemochory+hydrochory |
| *Rubus genevieri* | 2 | 1750 | 14 | 2.58 | Semi-woody | Shallow | Seeds and/or vegetatively | Fruits | Anemochory, hydrochory, anemochory+hydrochory |
| *Rubus idaeus* | 1 | 3487.5 | 80 | 1.7 | Semi-woody | Moderate | Seeds and/or vegetatively | Fruits | Zoochory |
| *Rubus lainzii* | 2 | 3250 | 14 | 2.58 | Semi-woody | Shallow | Seeds and/or vegetatively | Fruits | Anemochory, hydrochory, anemochory+hydrochory |
| *Rubus radula* | 2 | 2300 | 14 | 1.84 | Semi-woody | Shallow | Seeds and/or vegetatively | Fruits | Anemochory, hydrochory, anemochory+hydrochory |
| *Rubus ulmifolius* | 2 | 1460 | 14 | 2.52 | Semi-woody | Shallow | Seeds and/or vegetatively | Fruits | Zoochory |
| *Rubus vagabundus* | 2 | 2244 | 14 | 2.58 | Semi-woody | Shallow | Seeds and/or vegetatively | Fruits | Anemochory, hydrochory, anemochory+hydrochory |
| *Salix alba* | 25 | 800 | 48 | 0.12 | Woody | Shallow | Seeds | Seeds | Anemochory, hydrochory, anemochory+hydrochory |
| *Salix atrocinerea* | 12 | 992 | 48 | 0.001 | Woody | Shallow | Seeds and/or vegetatively | Seeds | Anemochory, hydrochory, anemochory+hydrochory |
| *Salix aurita* | 1.5 | 936.5 | 0 | 0.08 | Woody | Moderate | Seeds | Seeds | Anemochory, hydrochory, anemochory+hydrochory |
| *Salix caprea* | 10 | 900 | 0 | 0.25 | Woody | Shallow | Seeds | Seeds | Anemochory, hydrochory, anemochory+hydrochory |
| *Salix cinerea* | 1.2 | 1349 | 129.5 | 0.17 | Woody | Moderate | Seeds and/or vegetatively | Seeds | Anemochory, hydrochory, anemochory+hydrochory |
| *Salix glauca* | 2 | 489 | 148 | 0.16 | Woody | Moderate | Seeds and/or vegetatively | Seeds | Anemochory, hydrochory, anemochory+hydrochory |
| *Salix hastata* | 1 | 401 | 0 | 0.02 | Woody | Shallow | Seeds and/or vegetatively | Seeds | Anemochory, hydrochory, anemochory+hydrochory |
| *Salix herbacea* | 0.05 | 94.05 | 92.5 | 0.16 | Woody | Deep | Vegetativley | Seeds | Anemochory, hydrochory, anemochory+hydrochory |
| *Salix lanata* | 0.9 | 1133 | 0 | 0.08 | Woody | Moderate | Seeds | Seeds | Anemochory, hydrochory, anemochory+hydrochory |
| *Salix lapponum* | 2 | 294.05 | 92.5 | 0.07 | Woody | Deep | Seeds | Seeds | Anemochory, hydrochory, anemochory+hydrochory |
| *Salix myrsinites* | 0.6 | 323.24 | 74 | 0.34 | Woody | Deep | Seeds and/or vegetatively | Seeds | Anemochory, hydrochory, anemochory+hydrochory |
| *Salix myrtilloides* | 0.4 | 122.5 | 0 | 0.45 | Woody | Moderate | Seeds and/or vegetatively | Seeds | Anemochory, hydrochory, anemochory+hydrochory |
| *Salix neotricha* | 25 | 560 | 48 | 0.12 | Woody | Shallow | Seeds and/or vegetatively | Seeds | Anemochory, hydrochory, anemochory+hydrochory |
| *Salix pentandra* | 8 | 1011 | 96 | 0.14 | Woody | Shallow | Seeds | Seeds | Anemochory, hydrochory, anemochory+hydrochory |
| *Salix salviifolia* | 6 | 1200 | 48 | 0.001 | Woody | Shallow | Seeds and/or vegetatively | Seeds | Anemochory, hydrochory, anemochory+hydrochory |
| *Salix triandra* | 6 | 952.25 | 0 | 0.02 | Woody | Moderate | Seeds and/or vegetatively | Seeds | Anemochory, hydrochory, anemochory+hydrochory |
| *Sambucus nigra* | 5 | 11633 | 1 | 14 | Woody | Deep | Seeds and/or vegetatively | Fruits | Zoochory |
| *Sesamoides sufrruticosa* | 4.5 | 88.2 | 6 | 0.1 | Woody | Shallow | Seeds and/or vegetatively | Seeds | Zoochory |
| *Sorbus aucuparia* | 20 | 4000 | 6 | 7 | Woody | Moderate | Seeds | Fruits | Anemochory, hydrochory, anemochory+hydrochory |
| *Sorbus aucuparia* | 11.5 | 4000 | 6 | 7 | Woody | Moderate | Seeds | Fruits | Anemochory, hydrochory, anemochory+hydrochory |
| *Thymus mastichina* | 0.8 | 20.75 | 6 | 0.13 | Semi-woody | Moderate | Seeds | Fruits | Zoochory |
| *Ulex minor* | 1.5 | 5 | 1 | 3.2 | Semi-woody | Deep | Seeds | Seeds | Anemochory, hydrochory, anemochory+hydrochory |
| *Vaccinium microcarpum* | 0.35 | 6.3 | 2 | 0.22 | Semi-woody | Shallow | Seeds and/or vegetatively | Fruits | Anemochory, hydrochory, anemochory+hydrochory |
| *Vaccinium myrtillus* | 0.6 | 72 | 1 | 0.3 | Woody | Shallow | Seeds and/or vegetatively | Fruits | Zoochory |
| *Vaccinium myrtillus* | 0.5 | 72 | 1 | 0.3 | Woody | Shallow | Vegetativley | Fruits | Zoochory |
| *Vaccinium oxycoccos* | 0.5 | 15.5 | 4 | 0.48 | Semi-woody | Shallow | Vegetativley | Fruits | Anemochory, hydrochory, anemochory+hydrochory |
| *Vaccinium uliginosum* | 0.7 | 104 | 4 | 0.3 | Woody | Shallow | Seeds and/or vegetatively | Fruits | Zoochory |
| *Vaccinium vitis-idaea* | 0.17 | 48 | 4 | 0.3 | Woody | Shallow | Vegetativley | Fruits | Zoochory |
| *Vitis vinifera* subsp. *vinifera* | 35 | 8826 | 0.01 | 31 | Semi-woody | Moderate | Seeds and/or vegetatively | Seeds | Zoochory |

**Supplementary Table S3.** Mean, standard deviation (SD) and Range (max-min) of each trait in free-flowing and regulated sites, in Portugal and Sweden.

| PORTUGAL | | | | | | | | SWEDEN | | | | | |
| --- | --- | --- | --- | --- | --- | --- | --- | --- | --- | --- | --- | --- | --- |
|  | | Mean | | SD | | Range | | Mean | | SD | | Range | |
| Regime | | Free-flowing | Regulated | Free-flowing | Regulated | Free-flowing | Regulated | Free-flowing | Regulated | Free-flowing | Regulated | Free-flowing | Regulated |
| Canopy height | | 2.65 | 2.68 | 1.10 | 0.85 | 3.77 | 3.47 | 3.48 | 2.38 | 0.59 | 1.10 | 2.63 | 3.67 |
| Leaf area | | 394.66 | 410.76 | 183.47 | 188.63 | 726.91 | 736.79 | 421.59 | 287.11 | 173.39 | 178.82 | 637.34 | 678.46 |
| Seed bouyancy | | 127.77 | 147.30 | 52.52 | 31.69 | 169.07 | 161.54 | 179.94 | 163.00 | 68.90 | 75.21 | 241.00 | 226.00 |
| Seed weight | | 70.57 | 58.18 | 98.80 | 68.78 | 387.71 | 206.44 | 2.14 | 1.17 | 1.13 | 0.98 | 4.20 | 2.85 |
| Stem flexibility | Woody | 0.13 | 0.13 | 0.05 | 0.04 | 0.18 | 0.17 | 0.41 | 0.30 | 0.10 | 0.10 | 0.38 | 0.39 |
|  | Semi-woody | 0.04 | 0.05 | 0.02 | 0.02 | 0.06 | 0.06 | 0.05 | 0.03 | 0.02 | 0.03 | 0.07 | 0.09 |
| Rooting depth | Shallow | 0.08 | 0.09 | 0.03 | 0.02 | 0.11 | 0.10 | 0.03 | 0.04 | 0.02 | 0.02 | 0.05 | 0.05 |
|  | Moderate | 0.04 | 0.03 | 0.03 | 0.02 | 0.09 | 0.08 | 0.13 | 0.09 | 0.04 | 0.04 | 0.15 | 0.12 |
|  | Deep | 0.06 | 0.07 | 0.03 | 0.03 | 0.11 | 0.14 | 0.30 | 0.20 | 0.07 | 0.09 | 0.27 | 0.34 |
| Reproduction type | Seeds | 0.07 | 0.08 | 0.02 | 0.02 | 0.09 | 0.09 | 0.15 | 0.13 | 0.04 | 0.04 | 0.16 | 0.15 |
|  | Vegetatively | 0.00 | 0.00 | 0.01 | 0.00 | 0.02 | 0.02 | 0.11 | 0.07 | 0.03 | 0.05 | 0.11 | 0.16 |
|  | Seeds and/or vegetatively | 0.10 | 0.10 | 0.03 | 0.03 | 0.14 | 0.10 | 0.20 | 0.13 | 0.05 | 0.05 | 0.23 | 0.18 |
| Diaspore type | Seeds | 0.07 | 0.07 | 0.03 | 0.03 | 0.12 | 0.11 | 0.18 | 0.13 | 0.05 | 0.06 | 0.18 | 0.23 |
|  | Fruits | 0.10 | 0.10 | 0.04 | 0.03 | 0.12 | 0.09 | 0.26 | 0.18 | 0.07 | 0.06 | 0.28 | 0.25 |
| Dispersal vector | Anemochory, hydrochory, anemochory  +hydrochory | 0.08 | 0.09 | 0.03 | 0.02 | 0.12 | 0.08 | 0.30 | 0.23 | 0.07 | 0.09 | 0.25 | 0.36 |
|  | Zoochory | 0.09 | 0.08 | 0.04 | 0.03 | 0.13 | 0.14 | 0.16 | 0.10 | 0.05 | 0.04 | 0.20 | 0.16 |

**Supplementary Table S4**. Indicators of Hydrologic Alteration (IHA) used in the study, reflecting the regime characteristics i.e., magnitude, time, duration, frequency and rate of change, and their ecological relevance (adapted from Richter *et al*., 1996). Short names used are highlighted in bold.

| **Category** | Hydrological attribute (units) | **Ecological relevance** |
| --- | --- | --- |
| Mean value of monthly water variations | **January mean flow** (mm) | Magnitude of the water condition at any given time as a measure of the availability or suitability of habitat and defines wetted area or the position of a water table relative to wetland or riparian plant rooting zones. The timing of occurrence of particular water conditions can influence the degree of stress or mortality associated with extreme water conditions such as floods or droughts. |
|  | **February mean flow** (mm) |  |
|  | **March mean flow** (mm) |  |
|  | **April mean flow** (mm) |  |
|  | **May mean flow** (mm) |  |
|  | **June mean flow** (mm) |  |
|  | **July mean flow** (mm) |  |
|  | **August mean flow** (mm) |  |
|  | **September mean flow** (mm) |  |
|  | **October mean flow** (mm) |  |
|  | **November mean flow** (mm) |  |
|  | **December mean flow** (mm) |  |
| Duration of annual extreme water events | **1-day minimum**; annual minima, 1-day median (mm) | The duration of time over which a specific water condition exists may determine the degree to which stressful effects such as inundation or desiccation can accumulate. |
|  | **3-day minimum**; annual minima, 3-day median (mm) |  |
|  | **7-day minimum**; annual minima, 7-day median (mm) |  |
|  | **30-day minimum**; annual minima, 30-day median (mm) |  |
|  | **90-day minimum**; annual minima, 90-day median (mm) |  |
|  | **1-day maximum**; annual maxima, 1-day median (mm) |  |
|  | **3-day maximum**; annual maxima, 3-day median (mm) |  |
|  | **7-day maximum**; annual maxima, 7-day median (mm) |  |
|  | **30-day maximum**; annual maxima, 30-day median (mm) |  |
|  | **90-day maximum**; annual maxima, 90-day median (mm) |  |
|  | **Number of zero days**; number of days with no flow (mm) |  |
|  | **Base flow index** (=7-day minimum flow/average annual flow) |  |
| Frequencies of high/low water pulses | **Low pulse count**; number of low pulses (=mean of the number of low flow pulses each year) | The frequency of occurrence of specific water conditions such as droughts or floods may be tied to reproduction or mortality events, thereby influencing population dynamics. |
|  | **High pulse count**; number of high pulses (=mean number of high flow pulses each year) |  |
|  | **High pulse duration**; mean duration of high pulses (days) |  |
| Rate of change in water conditions | **Rise rate** (= median of all positive differences between consecutive daily values) (m^3^s^-1^) | The rate of change in water conditions may be tied to the ability of plant roots to maintain contact with phreatic water supplies. |
|  | **Fall rate** (=median of all negative differences between consecutive daily values) (m^3^s^-1^) |  |
|  | **Number of hydrologic reversals**; Number of hydrologic reversals (=mean of the number of flow variation between consecutive days) |  |

**Supplementary Table S5.** Analyses of variance (Two-way ANOVA) of Functional Richness and Functional Redundancy in flow regimes (free-flowing, regulated), in countries (Portugal and Sweden).

|  | **Df** | **SS** | **F** | **P-value** |
| --- | --- | --- | --- | --- |
| **Functional Richness** |  |  |  |  |
| Country | 1 | 0.377 | 80.861 | <0.001 |
| Regulation | 1 | 0.023 | 4.914 | 0.029 |
| Country:Regulation | 1 | 0.043 | 9.155 | 0.003 |
| **Functional Redundancy** |  |  |  |  |
| Country | 1 | 0.078 | 81.601 | <0.001 |
| Regulation | 1 | 0.007 | 7.194 | 0.009 |
| Country:Regulation | 1 | 0.001 | 1.126 | 0.291 |

**Supplementary Figure S1.** Residual analysis for the chosen models in Sweden: a) Functional Richness b) Functional Redundancy.


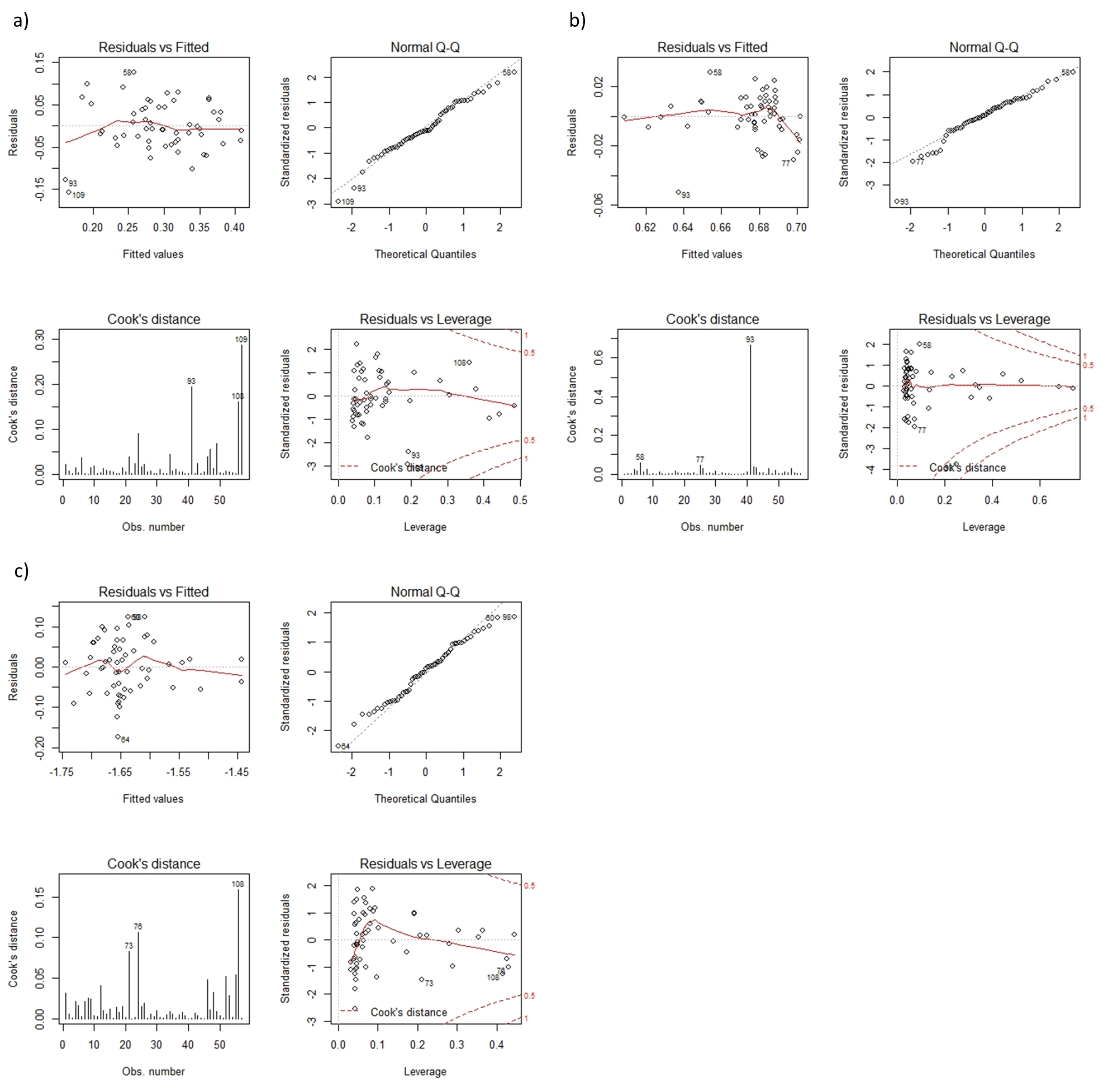


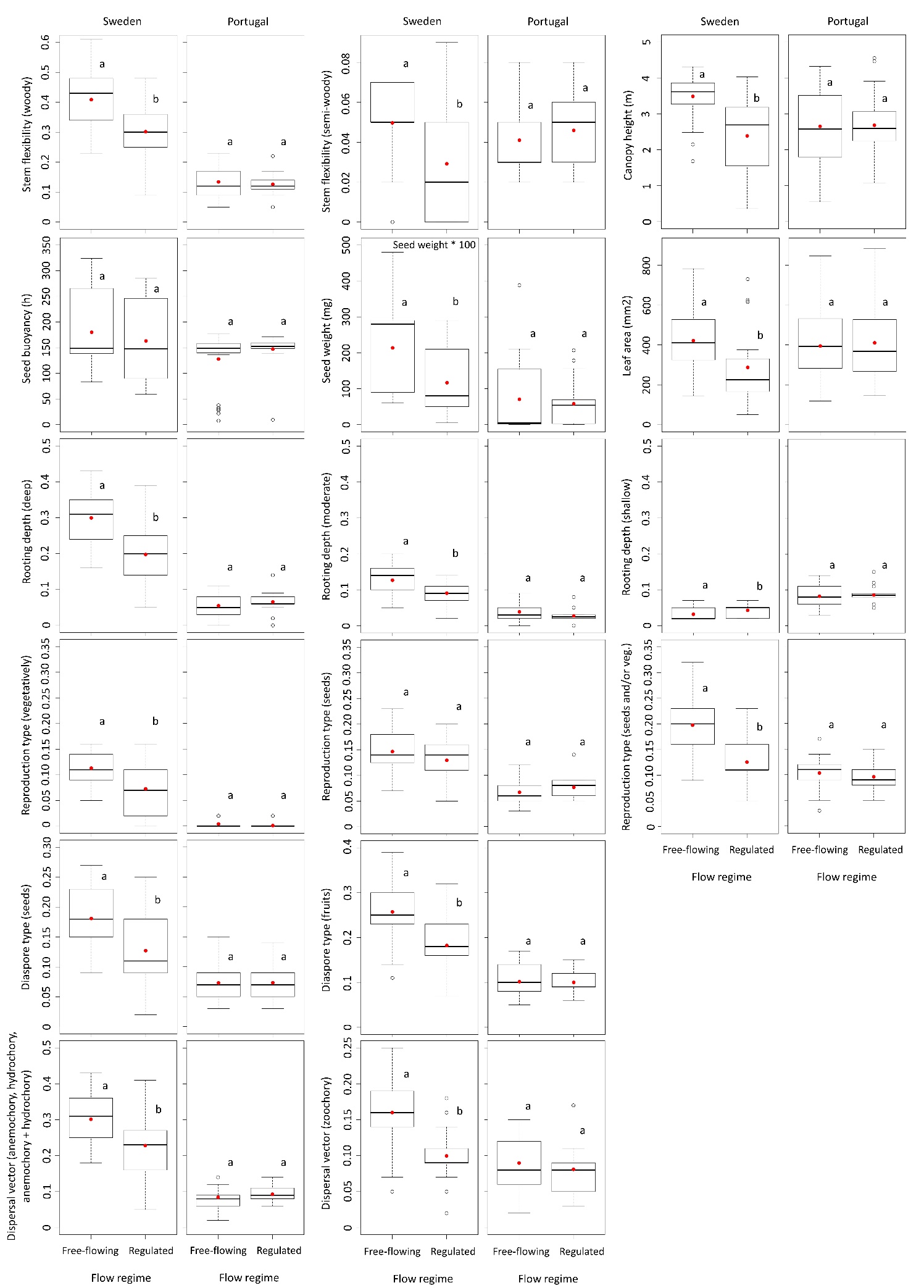


**Supplementary Figure S2.** Box-and-whisker plots for the selected traits (n=9) for the free-flowing and regulated sites in Sweden and Portugal. Letters identify the significantly different changes in trait values (p < 0.05). Traits with multiple categories (stem flexibility, rooting depth, reproduction type, diaspore type and dispersal vector) were counted as single trait.

**Supplementary Figure S3.** Functional diversity losses for Functional Richness and Functional Redundancy in Portugal and Sweden. Values at the y-axes represent mean differences in indices values between regulated and free-flowing sites.


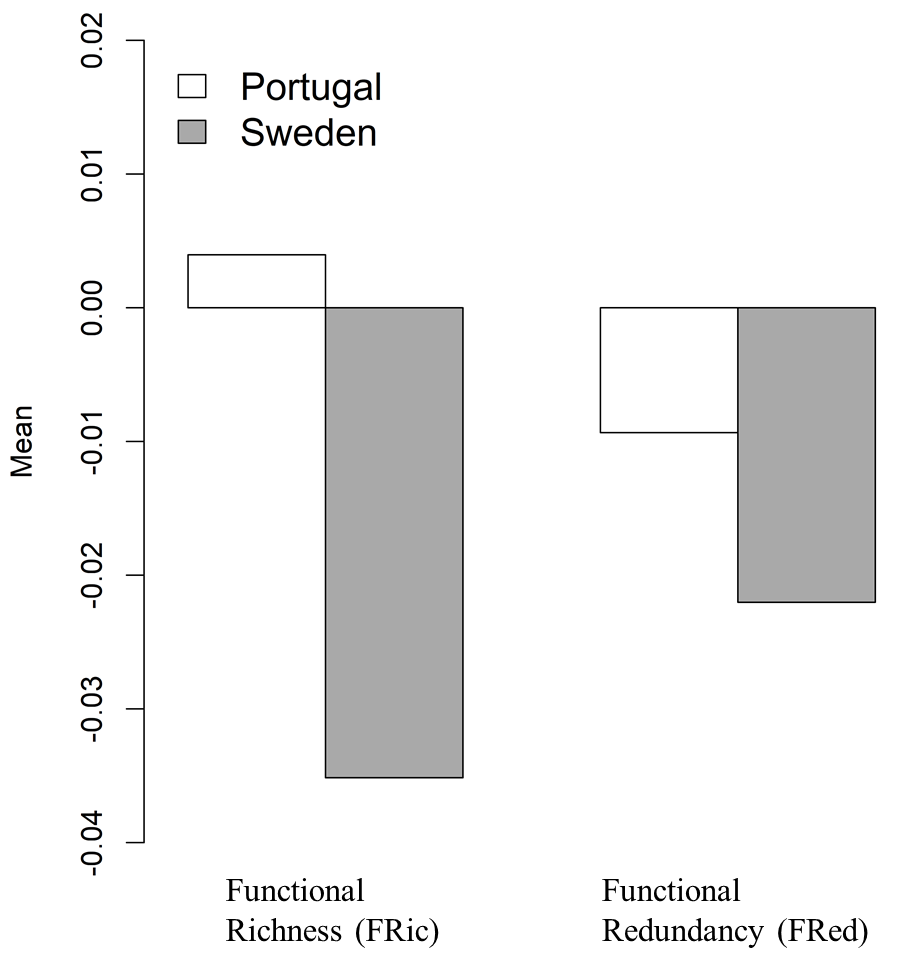


**Supplementary Table S6. Number of sampling sites (n).** Mean, standard deviation (SD), and Range (max-min) for Functional Richness and Functional Redundancy in free-flowing and regulated sites, in Portugal and Sweden.

|  |  | **n** | **Mean** | **SD** | **Range** |
| --- | --- | --- | --- | --- | --- |
| **Portugal** | **Functional Richness** |  |  |  |  |
|  | Free-flowing | 30 | 0.033 | 0.020 | 0.075 |
|  | Regulated | 22 | 0.037 | 0.023 | 0.067 |
|  | **Functional Redundancy** |  |  |  |  |
|  | Free-flowing | 30 | 0.408 | 0.035 | 0.173 |
|  | Regulated | 22 | 0.399 | 0.021 | 0.075 |
|  |  |  |  |  |  |
| **Sweden** | **Functional Richness** |  |  |  |  |
|  | Free-flowing | 32 | 0.109 | 0.040 | 0.142 |
|  | Regulated | 25 | 0.074 | 0.038 | 0.153 |
|  | **Functional Redundancy** |  |  |  |  |
|  | Free-flowing | 32 | 0.467 | 0.021 | 0.078 |
|  | Regulated | 25 | 0.445 | 0.041 | 0.149 |

**Supplementary Table S7.** Analyses of variance (Two-way ANOVA) of river hydrological attributes (Indices of Hydrologic Alteration, IHA). Factors are Regulation (free-flowing and regulated), and Country (Portugal and Sweden).

| **Hydrological attribute** | **Df** | **SS** | **F** | **P-value of F-tests of principal and interaction effects** |
| --- | --- | --- | --- | --- |
| **January mean flow** |  |  |  |  |
| Country | 1 | 177442 | 29.97 | <0.001 |
| Regulation | 1 | 144673 | 24.44 | <0.001 |
| Country:Regulation | 1 | 157582 | 26.62 | <0.001 |
| **February mean flow** |  |  |  |  |
| Country | 1 | 168588 | 25.64 | <0.001 |
| Regulation | 1 | 163880 | 24.92 | <0.001 |
| Country:Regulation | 1 | 180473 | 27.45 | <0.001 |
| **March mean flow** |  |  |  |  |
| Country | 1 | 145795 | 24.90 | <0.001 |
| Regulation | 1 | 130268 | 22.25 | <0.001 |
| Country:Regulation | 1 | 147246 | 25.15 | <0.001 |
| **April mean flow** |  |  |  |  |
| Country | 1 | 168458 | 32.11 | <0.001 |
| Regulation type | 1 | 74635 | 14.23 | <0.001 |
| Country:Regulation | 1 | 84276 | 16.06 | <0.001 |
| **May mean flow** |  |  |  |  |
| Country | 1 | 1093771 | 34.597 | <0.001 |
| Regulation | 1 | 11433 | 0.362 | 0.549 |
| Country:Regulation | 1 | 6510 | 0.206 | 0.651 |
| **June mean flow** |  |  |  |  |
| Country | 1 | 1523872 | 53.827 | <0.001 |
| Regulation | 1 | 5088 | 0.180 | 0.672 |
| Country:Regulation | 1 | 3504 | 0.124 | 0.726 |
| **July mean flow** |  |  |  |  |
| Country | 1 | 992260 | 67.892 | <0.001 |
| Regulation | 1 | 2988 | 0.204 | 0.652 |
| Country:Regulation | 1 | 2805 | 0,192 | 0.662 |
| **August mean flow** |  |  |  |  |
| Country | 1 | 649237 | 66.395 | <0.001 |
| Regulation | 1 | 34246 | 3.502 | 0.0641 |
| Country:Regulation | 1 | 31154 | 3.186 | 0.0772 |
| **September mean flow** |  |  |  |  |
| Country | 1 | 540514 | 63.232 | <0.001 |
| Regulation | 1 | 43586 | 5.099 | 0.026 |
| Country:Regulation | 1 | 39916 | 4.670 | 0.033 |
| **October mean flow** |  |  |  |  |
| Country | 1 | 428202 | 61.670 | <0.001 |
| Regulation | 1 | 44951 | 6.474 | 0.0124 |
| Country:Regulation | 1 | 45094 | 6.494 | 0.0123 |
| **November mean flow** |  |  |  |  |
| Country | 1 | 274172 | 47.74 | <0.001 |
| Regulation | 1 | 74105 | 12.90 | <0.001 |
| Country:Regulation | 1 | 86896 | 15.13 | <0.001 |
| **December mean flow** |  |  |  |  |
| Country | 1 | 210021 | 37.69 | <0.001 |
| Regulation | 1 | 108674 | 19.50 | <0.001 |
| Country:Regulation | 1 | 129887 | 23.31 | <0.001 |
| **1-day minimum** |  |  |  |  |
| Country | 1 | 22240 | 23.096 | <0.001 |
| Regulation | 1 | 4655 | 4.834 | 0.030 |
| Country:Regulation | 1 | 4209 | 4.370 | 0.039 |
| **3-day minimum** |  |  |  |  |
| Country | 1 | 27029 | 22.715 | <0.001 |
| Regulation | 1 | 7408 | 6.226 | 0.014 |
| Country:Regulation | 1 | 6829 | 5.740 | 0.018 |
| **7-day minimum** |  |  |  |  |
| Country | 1 | 34360 | 22.214 | <0.001 |
| Regulation | 1 | 12084 | 7.812 | 0.006 |
| Country:Regulation | 1 | 11334 | 7.327 | 0.007 |
| **30-day minimum** |  |  |  |  |
| Country | 1 | 50327 | 23.60 | <0.001 |
| Regulation | 1 | 24006 | 11.26 | 0.001 |
| Country:Regulation | 1 | 22568 | 10.58 | 0.002 |
| **90-day minimum** |  |  |  |  |
| Country | 1 | 84316 | 28.38 | <0.001 |
| Regulation | 1 | 48224 | 16.13 | <0.001 |
| Country:Regulation | 1 | 44914 | 15.12 | <0.001 |
| **1-day maximum** |  |  |  |  |
| Country | 1 | 3899340 | 33.753 | <0.001 |
| Regulation | 1 | 135582 | 1.174 | 0.281 |
| Country:Regulation | 1 | 17877 | 0.155 | 0.695 |
| **3-day maximum** |  |  |  |  |
| Country | 1 | 4212866 | 38.584 | <0.001 |
| Regulation | 1 | 90552 | 0.829 | 0.365 |
| Country:Regulation | 1 | 3579 | 0.033 | 0.857 |
| **7-day maximum** |  |  |  |  |
| Country | 1 | 4128598 | 42.952 | <0.001 |
| Regulation | 1 | 48518 | 0.505 | 0.479 |
| Country:Regulation | 1 | 430 | 0.004 | 0.947 |
| **30-day maximum** |  |  |  |  |
| Country | 1 | 2676619 | 50.336 | <0.001 |
| Regulation | 1 | 2216 | 0.042 | 0.839 |
| Country:Regulation | 1 | 6573 | 0.124 | 0.726 |
| **90-day maximum** |  |  |  |  |
| Country | 1 | 1473247 | 56.550 | <0.001 |
| Regulation | 1 | 7270 | 0.279 | 0.598 |
| Country:Regulation | 1 | 24828 | 0.953 | 0.331 |
| **Number of zero days** |  |  |  |  |
| Country | 1 | 108013 | 15.369 | <0.001 |
| Regulation | 1 | 30334 | 4.316 | 0.040 |
| Country:Regulation | 1 | 24312 | 3.459 | 0.066 |
| **Base flow index** |  |  |  |  |
| Country | 1 | 0.329 | 10.080 | 0.002 |
| Regulation | 1 | 0.377 | 11.545 | <0.001 |
| Country:Regulation | 1 | 0.090 | 2.761 | 0.100 |
| **Low pulse count** |  |  |  |  |
| Country | 1 | 1304 | 19.513 | <0.001 |
| Regulation | 1 | 99 | 1.484 | 0.226 |
| Country:Regulation | 1 | 603 | 9.029 | 0.003 |
| **High pulse count** |  |  |  |  |
| Country | 1 | 954.1 | 38.504 | <0.001 |
| Regulation | 1 | 26.1 | 1.053 | 0.307 |
| Country:Regulation | 1 | 62.7 | 2.531 | 0.115 |
| **High pulse duration** |  |  |  |  |
| Country | 1 | 9355 | 142.662 | <0.001 |
| Regulation | 1 | 442 | 6.735 | 0.011 |
| Country:Regulation | 1 | 343 | 5.227 | 0.024 |
| **Rise rate** |  |  |  |  |
| Country | 1 | 3225 | 25.992 | <0.001 |
| Regulation | 1 | 912 | 7.353 | 0.008 |
| Country:Regulation | 1 | 348 | 2.803 | 0.010 |
| **Fall rate** |  |  |  |  |
| Country | 1 | 1405 | 14.473 | <0.001 |
| Regulation | 1 | 1474 | 15.182 | <0.001 |
| Country:Regulation | 1 | 919 | 9.468 | 0.003 |
| **Number of hydrologic reversals** |  |  |  |  |
| Country | 1 | 45107 | 20.176 | <0.001 |
| Regulation | 1 | 5106 | 2.284 | 0.134 |
| Country:Regulation | 1 | 149790 | 66.999 | <0.001 |

**Supplementary Table S8.** Post-Hoc Test (TukeyHSD) of river hydrological attributes in flow regimes free-flowing (FF) and regulated (REG), in countries Portugal (PT) and Sweden (SW). The hydrological attributes are defined in Supplementary Table S4. This table presents the sign of the differences between means of each hydrological attribute for each pair of factor levels. Non-significant differences are denoted by “–“; positive and negative differences are denoted by “>0” and “<0”, respectively.

| **Hydrological attribute** | **SW:FF - PT:FF** | **PT:REG - PT:FF** | **SW:REG - PT:FF** | **PT:REG - SW:FF** | **SW:REG - SW:FF** | **SW:REG-PT:REG** |
| --- | --- | --- | --- | --- | --- | --- |
| January mean flow | - | - | > 0 | - | > 0 | > 0 |
| February mean flow | - | - | > 0 | - | > 0 | > 0 |
| March mean flow | - | - | > 0 | - | > 0 | > 0 |
| April mean flow | - | - | > 0 | - | > 0 | > 0 |
| May mean flow | > 0 | - | > 0 | < 0 | - | > 0 |
| June mean flow | > 0 | - | > 0 | < 0 | - | > 0 |
| July mean flow | > 0 | - | > 0 | < 0 | - | > 0 |
| August mean flow | > 0 | - | > 0 | < 0 | - | > 0 |
| September mean flow | > 0 | - | > 0 | < 0 | > 0 | > 0 |
| October mean flow | > 0 | - | > 0 | < 0 | > 0 | > 0 |
| November mean flow | > 0 | - | > 0 | < 0 | > 0 | > 0 |
| December mean flow | - | - | > 0 | - | > 0 | > 0 |
| 1-day minimum | - | - | > 0 | - | > 0 | > 0 |
| 3-day minimum | - | - | > 0 | - | > 0 | > 0 |
| 7-day minimum | - | - | > 0 | - | > 0 | > 0 |
| 30-day minimum | - | - | > 0 | - | > 0 | > 0 |
| 90-day minimum | - | - | > 0 | - | > 0 | > 0 |
| 1-day maximum | > 0 | - | > 0 | < 0 | - | > 0 |
| 3-day maximum | > 0 | - | > 0 | < 0 | - | > 0 |
| 7-day maximum | > 0 | - | > 0 | < 0 | - | > 0 |
| 30-day maximum | > 0 | - | > 0 | < 0 | - | > 0 |
| 90-day maximum | > 0 | - | > 0 | < 0 | - | > 0 |
| Number of zero days | - | > 0 | - | > 0 | - | < 0 |
| Base flow index | > 0 | > 0 | > 0 | - | - | - |
| Low pulse count | < 0 | < 0 | < 0 | - | - | - |
| High pulse count | < 0 | - | < 0 | > 0 | - | < 0 |
| High pulse duration | > 0 | - | > 0 | < 0 | < 0 | > 0 |
| Rise rate | > 0 | - | > 0 | - | > 0 | > 0 |
| Fall rate | - | - | < 0 | - | < 0 | < 0 |
| Number of reversals | < 0 | < 0 | < 0 | - | > 0 | > 0 |

**Supplementary Table S9.** References used in the **Table 1** (Legacy effects which influence riparian woody vegetation in the Mediterranean and boreal biomes).

ACIA 2005. Arctic Climate Impact Assessment. ACIA overview repost. Cambridge University Press. 1020.

Aguiar FC, Ferreira MT. 2005. Human-disturbed landscapes: effects on composition and and integrity of riparian woody vegetation in the Tagus River basin, Portugal. Environmental Conservation 32:30–41.

Aguiar FC, Ferreira MT, Albuquerque A. 2006. Patterns of exotic and native plant species richness and cover along a semi-arid Iberian river and across its floodplain. Plant Ecology 184:189 –202.

Aguiar FC, Cerdeira JO, Martins MJ, Ferreira MT. 2013. Riparian forests of Southwest Europe: are functional trait and species composition assemblages constrained by environment? Journal of Vegetation Science 24:628–638.

Anisimov OA, Nelson FE. 1996. Permafrost distribution in the Northern Hemisphere under scenarios of climatic changes. Ambio 40:17–31.

Astrom M, Aaltonen EK, Koivusaari J. 2001. Effect of ditching operations on stream-water chemistry in a boreal forested catchment. Science of Total Environment 279:117–29.

Callaghan TV, Johansson M, Brown RD, Groisman PY, Labba N, Radionov V, Barry RG, Bulygina ON, Essery RLH, Frolov DM, Golubev VN, Grenfell TC, Petrushina MN, Razuvaev VN, Robinson DA, Romanov P, Shindell D, Shmakin AB, Sokratov SA, Warren S, Yang D. 2011. The changing face of Arctic snow cover: a synthetis of observed and projected change. Global and Planetary Change 14:59–72.

Chesworth W. 2008. Encyclopedia of soil science. Dordrecht: Springer.

Ferreira MT, Albuquerque A, Aguiar FC, Catarino LF. 2002. Seasonal and yearly variations of macrophytes in a Southern Iberian river. Proceedings Internationale Vereinigung fur teoretische und angewandte limnologie: verhandlungen 27:3833–3837.

Gasith A, Resh VH. 1999. Streams in Mediterranean climate regions: abiotic influences and biotic responses to predictable seasonal events. Annual Review of Ecology and Systematics 30:51–81.

Gonçalves Jr JF, Graça MAS, Callisto M. 2006. Leaf-litter breakdown in 3 streams in temperate, Mediterranean, and tropical Cerrado climates. Journal of North American Benthological Society 25:344–355.

Grabs T, Bishop K, Laudon H, Lyon SW, Seibert J. 2012. Riparian zone hydrology and soil water total organic carbon (TOC): implications for spatial variability and upscaling of lateral riparian TOC exports. Biogeosciences 9:3901–16.

Hooke JM. 2006. Human impacts on fluvial systems in the Mediterranean region. Geomorphology 79:311–335.

Kondolf GM, Batalla RJ. 2005. Hydrological effects of dams and water diversions on rivers of Mediterranean-climate regions: examples from California. Developements in Earth and Surface Processes 7:197–211.

Köppen W. 1931. Klimakarte der Erde. Grundriss der Klimakunde, 2nd Ed., Berlin and Leipzig.

Ledesma JLJ, Futter MN, Blackburn M, Lidman F, Grabs T, Sponseller RA, Laudon H, Bishop KH, Kohler SJ. 2018. Towards an improved conceptualization of riparian zones in boreal forest headwaters. Ecosystems 21:297–315.

Loidi J. 2018. Plant eco-morphological traits as adaptations to environmental conditions: some comparisons between different biomes across the World. Geographical Changes in Vegetation and Plant Functional Types pp.59-71.

Lupon A, Bernal S, Poblador S, Marti E, Sabater F. 2016. The influence of riparian evapotranspiration on stream hydrology and nitrogen retention in a subhumid Mediterranean catchment. Hydrological Earth System Science 20:3831–42.

Magdaleno F, Fernández JA. 2011. Hydromorphological alteration of a large Mediterranean river: relative role of high and low flows on the evolution of riparian forests and channel morphology. River Research Applications 27:374–387.

Myers N, Mittermeier RA, Mittermeier CG, da Fonseca GAB, Kent J. 2000. Biodiversity hotspots for conservation priorities. Nature 403:853–858.

Nilsson C, Polvi LE, Lind L. 2015. Extreme events in streams and rivers in arctic and subarctic regions in an uncertain future. Freshwater Biology 60:2535–2546.

Prowse T, Alfredsen K, Beltaos S, Bonsal BR, Duguay C, Korhola A. McNamara J, Pienitz R, Vincent WF, Vuglinsky V, Weyhnmeyer GA. 2011. Past and future changes in arctic lake and river ice. Ambio 40:53–62.

Sabater S, Elosegi A, Acuña V, Basaguren A, Muñoz I, Pozo J. 2008. Effect of climate on the trophic structure of temperate forested streams. A comparison of Mediterranean and Atlantic streams. Science of Total Environment 390:475–484.

Santos MJ. 2010. Encroachment of upland Mediterranean plant species in riparian ecosystems of southern Portugal. Biodiversity Conservation 19:2667–84.

Vallee S, Payette S. 2007. Collapse of permafrost mounds along a subarctic river over the last 100 years (northern Quebec). Geomorphology 90:162–170.

Wrona FJ, Prowse TD, Reist JD, Hobbie JE, Levesque LMJ, Vincent WF. 2006. Climate change effects on aquatic biota, ecosystem structure and function. Ambio 35:359–369.

Yaalon DH. 1997. Soils in the Mediterranean region: what makes them different? Catena 28:157-169.
